# Association of expression of epigenetic molecular factors with DNA methylation and sensitivity to chemotherapeutic agents in cancer cell lines

**DOI:** 10.1186/s13148-021-01026-4

**Published:** 2021-03-06

**Authors:** Suleyman Vural, Alida Palmisano, William C. Reinhold, Yves Pommier, Beverly A. Teicher, Julia Krushkal

**Affiliations:** 1 Biometric Research Program, Division of Cancer Treatment and Diagnosis, National Cancer Institute, 9609 Medical Center Dr., Rockville, MD 20850 USA; 2 General Dynamics Information Technology (GDIT), 3150 Fairview Park Drive, Falls Church, VA 22042 USA; 3 Developmental Therapeutics Branch, Center for Cancer Research, National Cancer Institute, NIH, Bethesda, MD 20892 USA; 4 Molecular Pharmacology Program, Division of Cancer Treatment and Diagnosis, National Cancer Institute, Bethesda, MD 20892 USA

**Keywords:** DNA methylation, Gene expression, Epigenetic analysis, Cancer drug treatment

## Abstract

**Background:**

Altered DNA methylation patterns play important roles in cancer development and progression. We examined whether expression levels of genes directly or indirectly involved in DNA methylation and demethylation may be associated with response of cancer cell lines to chemotherapy treatment with a variety of antitumor agents.

**Results:**

We analyzed 72 genes encoding epigenetic factors directly or indirectly involved in DNA methylation and demethylation processes. We examined association of their pretreatment expression levels with methylation beta-values of individual DNA methylation probes, DNA methylation averaged within gene regions, and average epigenome-wide methylation levels. We analyzed data from 645 cancer cell lines and 23 cancer types from the Cancer Cell Line Encyclopedia and Genomics of Drug Sensitivity in Cancer datasets. We observed numerous correlations between expression of genes encoding epigenetic factors and response to chemotherapeutic agents. Expression of genes encoding a variety of epigenetic factors, including *KDM2B, DNMT1, EHMT2, SETDB1, EZH2, APOBEC3G,* and other genes, was correlated with response to multiple agents. DNA methylation of numerous target probes and gene regions was associated with expression of multiple genes encoding epigenetic factors, underscoring complex regulation of epigenome methylation by multiple intersecting molecular pathways. The genes whose expression was associated with methylation of multiple epigenome targets encode DNA methyltransferases*,* TET DNA methylcytosine dioxygenases*,* the methylated DNA-binding protein ZBTB38, KDM2B, SETDB1, and other molecular factors which are involved in diverse epigenetic processes affecting DNA methylation. While baseline DNA methylation of numerous epigenome targets was correlated with cell line response to antitumor agents, the complex relationships between the overlapping effects of each epigenetic factor on methylation of specific targets and the importance of such influences in tumor response to individual agents require further investigation.

**Conclusions:**

Expression of multiple genes encoding epigenetic factors is associated with drug response and with DNA methylation of numerous epigenome targets that may affect response to therapeutic agents. Our findings suggest complex and interconnected pathways regulating DNA methylation in the epigenome, which may both directly and indirectly affect response to chemotherapy.

## Background

Cancer cells acquire multiple epigenomic alterations, including aberrant DNA methylation and DNA hydroxymethylation of genes and genome regions, loss or gain of imprinting and allele switching of imprinted loci, and global DNA hypomethylation [1–6]. Epigenetic changes in malignant cells result in transcriptional and post-transcriptional rewiring, influencing cell cycle, growth, and proliferation. Epigenetic dysregulation in tumors leads to silencing of tumor suppressor genes and of genes involved in DNA repair, activates oncogene expression, alters gene function, affects transcriptional regulatory networks, and increases genome instability [1, 4, 6–11]. Global DNA hypomethylation of malignant cells has been associated with tumor evasion of the immune response [12].

Many epigenetic factors directly or indirectly dynamically influence genome region-specific or global DNA methylation in the germ line, embryonic, or somatic adult cells (Additional file 1: Table S1). Below, we refer to their genes as GMDs (genes affecting DNA methylation or demethylation). Products of the DNA methyltransferase (DNA 5′ cytosine-methyltransferase, or DNMT) genes *DNMT1*, *DNMT3A* and *DNMT3B* are directly involved in DNA methylation. Products of TET methylcytosine dioxygenase genes (*TET1, TET2* and *TET3*) and products of *AICDA* (*AID*) and *APOBEC*, *TDG*, *MBD4*, *SMUG1,* and *GADD45A* participate in DNA demethylation through DNA hydroxymethylation, deamination, base excision repair (BER), and other mechanisms [4, 13–19]. For example, a molecular complex containing AID, TDG, and GADD45A participates in DNA demethylation via the BER pathway [20].

Many factors participate in molecular complexes that affect DNA methylation or demethylation, participate in methylation-dependent targeting of other molecular factors to genome regions, or regulate binding and/or activities of DNMTs, TETs, and other epigenetic factors, either directly or via intermediate metabolites. Examples include MBD1, MBD2, MBD3, MBD4, PCNA, USP7 (HAUSP), DNMT3L, UHRF1, UHRF2, DMAP1, ZBTB4, ZBTB33 (KAISO), ZBTB38, RBPJ, G9A (EHMT2), KAT5 (TIP60), SUV39H1, HDAC1, SIRT1, EZH2, CSNK1D, CSNK1E, and SUMO1 (Additional file 1: Table S1) [4, 13, 21–29]. *IDH1* and *IDH2* mutations lead to overproduction of cellular metabolites which interfere with TET-mediated conversion of 5-methylcytosine (5-mC) to 5-hydroxymethylcytosine (5-hmC) [30]. GLP (EHMT1), G9A (EHMT2), ZFP57, DPPA3 (PGC7, or STELLA), TRIM28 (KAP1), SETDB1, DNMT3L, EED, EZH2, SUZ12, and ZSCAN4 are involved in regulation, de novo methylation, and/or maintenance of imprinted regions and/or affect DNA methylation in embryonic stem cells [18, 27, 31–36].

While many GMDs are involved in methylation or demethylation of 5-mC, MGMT demethylates O^6^-methylguanine (O^6^-meG) lesions and removes O^6^-alkyl adducts, whereas ALKBH2 and ALKBH3 demethylate DNA via removal of 1-methyladenine (N^1^-meA) and 3-methylcytosine (N^3^-meC) [37–39].

A number of epigenetic factors have complex and intertwined roles affecting DNA methylation. There is an extensive cross-talk among the DNA methylation, demethylation, and histone modification pathways in germ line, embryonic stem, normal somatic, and malignant cells [26, 27, 31, 35, 40–42]. DNA methylation is influenced by histone modifications, and histone methylation and acetylation marks directly affect DNMT localization, binding, and activities [27, 35, 40, 43]. Specific GMD roles in DNA methylation and demethylation and examples of their interactions are presented in Additional file 1: Table S1 and accompanying text.

GMD components may directly or indirectly affect sensitivity of cancer cells to treatment. DNMTs are directly inhibited by DNA hypomethylating agents, while other antitumor agents target additional GMD products [1, 7, 44–49]. The Hsp90 inhibitor 17-DMAG diminishes the binding of DNMT1 and of the histone methyltransferase EZH2 to Hsp90, attenuates the interaction between DNMT1 and EZH2, and mediates the depletion of DNMT1 and EZH2 [50]. HDAC inhibitors (HDACi) affect DNA methylation through a variety of mechanisms. Vorinostat downregulates transcription of *DNMT1* and *DNMT3B* and changes DNA methylation of *TERT* and *DLC1* [51–53]. Panobinostat depletes protein levels of DNMT1 and EZH2 and disrupts DNMT1 interaction with EZH2 and the polycomb repressive complex 2 (PRC2) [50]. Trichostatin A downregulates gene and protein expression of DNMT1 and induces global DNA hypomethylation [54]. Belinostat reduces global DNA methylation and depletes protein levels of the PRC2 subunits EZH2 and SUZ12 [55].

Among examples of the influence of DNA methylation on tumor sensitivity to treatment, *MGMT* promoter methylation downregulates *MGMT* expression, disrupting MGMT role in DNA repair, which is linked to resistance to nitrosourea-based antitumor agents, temozolomide, and radiation [37, 56, 57]. Specific DNA methylation patterns or methylation of individual genes have been associated with resistance to different types of cancer drugs, e.g., the platinum compound cisplatin, poly(ADP-ribose) polymerase (PARP) inhibitors, the microtubule-disrupting agent paclitaxel, and the cytidine analog cytarabine [11, 57–66], which may suggest indirect influences of GMD on drug response.

**Fig. 1:**
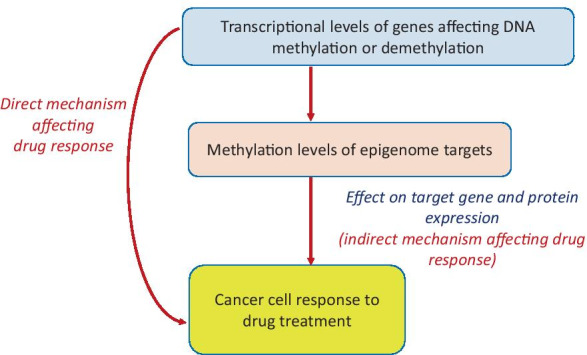
Possible hypothetical mechanisms by which GMD expression may directly or indirectly affect response of cancer cells drugs. GMDs may directly influence drug response through a variety of mechanisms. Among indirect influences of GMDs on drug response examined in this study, we focused on the effect of GMD expression on DNA methylation of epigenome targets

Due to the significance of epigenetic factors in regulation of DNA methylation, it is important to investigate how GMD expression may directly or indirectly affect tumor response to treatment (Fig. 1). We used cancer cell line data from two public resources, the Cancer Cell Line Encyclopedia (CCLE) and the Genomics of Drug Sensitivity in Cancer [67–72], to examine associations of drug response with 72 GMDs (Additional file 1: Table S1) that are directly or indirectly involved in DNA methylation or demethylation. We investigated correlations of their pretreatment expression with methylation of their putative genome targets and with cancer cell line response to a variety of antitumor agents with different mechanisms of action.

## Methods

### Selection of candidate genes involved in DNA methylation and demethylation

Additional file 1: Table S1 provides the list of the 72 GMDs analyzed in this study. Their products are directly or indirectly involved in DNA methylation or demethylation in human tissues. Information about their biological roles in DNA methylation or demethylation was obtained from the biomedical literature and from GeneCards [73] and the Online Mendelian Inheritance in Men (OMIM) [74].

### Drug response data

To examine the relationship between pretreatment GMD expression and tumor response to antitumor agents, we used gene expression, DNA methylation, and drug response data for 645 cell lines, the identity of which was matched between the Cancer Cell Line Encyclopedia and the Genomics of Drug Sensitivity in Cancer datasets [67–72] (Additional file 2: Table S2). The IC50 measures of drug response, representing the total drug concentration that reduced cell activity by 50%, were available for 24 agents from CCLE [67, 68, 72]. Additional IC50 values for 251 agents were obtained from the Genomics of Drug Sensitivity in Cancer portal [69, 71, 75]. Below were refer to these drug response measures as GDSC measures. After our analysis was completed, GDSC released a second batch of drug response values, referring in their release to the initial dataset as GDSC1 and the second dataset as GDSC2 [75]. All GDSC data analyzed in our study were from the GDSC1 dataset.

All CCLE and GDSC drug sensitivity values were transformed to the log_10_(IC50) scale. Cell line identities in the CCLE and GDSC datasets were verified using Cellosaurus [76]. Response measures for 11 agents which were present in both CCLE and GDSC data were analyzed separately, without combining the CCLE and GDSC measures. For those agents in the GDSC dataset that had duplicate measurements [71], we used the combined average of their drug response measures from separate experiments. The resulting dataset included 275 CCLE and GDSC drug response measures for 255 distinct antitumor agents. The concordance of drug response measures between the CCLE and GDSC datasets has been reported previously [77–79]. Information about mechanisms of action of the agents was collected from the CCLE and GDSC portals, their accompanying publications [67, 68, 71, 75] and biomedical literature.

### Gene expression data retrieval

For the RNA-seq data used in this project, RPKM gene expression values were downloaded from the CCLE portal of the Broad Institute [72, 80]. RNA sample library preparation using Illumina TruSeq RNA Sample Preparation protocol, RNA-sequencing using Illumina HiSeq 2000 and HiSeq 2500, and initial data processing was previously described by the CCLE project [81].

### DNA methylation data filtering

Cell line methylation data for 485,512 probes, generated by the GDSC project [71] using Illumina Infinium HumanMethylation450 (450 K) BeadChip array (Illumina, Inc.), were downloaded from NCBI GEO [82]. Methylation probe beta-values for individual cell lines with detection *p-*values ≥ 10^–3^ and 340 entire probes with median detection *p-*values ≥ 10^–6^ were excluded. In addition, 60,332 probes overlapping with single nucleotide polymorphisms were filtered out based on the probe masking recommendations for hg19 (GRCh37) [83, 84]. The final methylation dataset used in analysis had methylation beta-values for 424,840 probes that passed all filtering. Chromosomal regions (cytobands) were identified according to the UCSC genome annotation for the hg19 (GRCh37) human genome assembly based on the probe coordinates in the Illumina Infinium HumanMethylation 450 K BeadChip annotation.

### Calculation of gene region-averaged methylation values

In order to compute gene region-averaged methylation beta-values from individual probe measures, we developed an R program (available upon request) which followed the algorithm developed previously by the authors of the IMA software [85]. We recently reported a version of our software adapted for the Illumina Infinium MethylationEPIC BeadChip array [86]. For this study, we used a similar version which we adapted for the Illumina Infinium HumanMethylation450 BeadChip array. We used the Illumina Infinium HumanMethylation450 BeadChip annotation of each probe [87] according to the UCSC genome browser data to compute gene region-averaged methylation for 6 gene regions: TSS1500 (200–1500 bases upstream of the transcriptional start site, or TSS), TSS200 (0–200 bases upstream of the TSS), 5′UTR (within the 5′ untranslated region, between the TSS and the ATG start site), 1st exon, gene body (between the ATG start site and the stop codon), and 3′UTR (within the 3′ untranslated region, between the stop codon and poly A signal). The resulting methylation values were computed for 93,591 regions in 20,643 genes and ncRNA, with each gene represented by up to 6 regions. Additional file 20: Fig. S1 shows the distribution of methylation beta-values among 424,840 individual probes, the combined distribution among 93,591 gene regions, and separate distributions for each gene region category (TSS1500, TSS200, 5′UTR, 1st exon, gene body, and 3′ UTR) in 645 cell lines.

### Association analysis of GMD expression, epigenome-wide methylation of individual probes and gene regions, and drug response

**Fig. 2:**
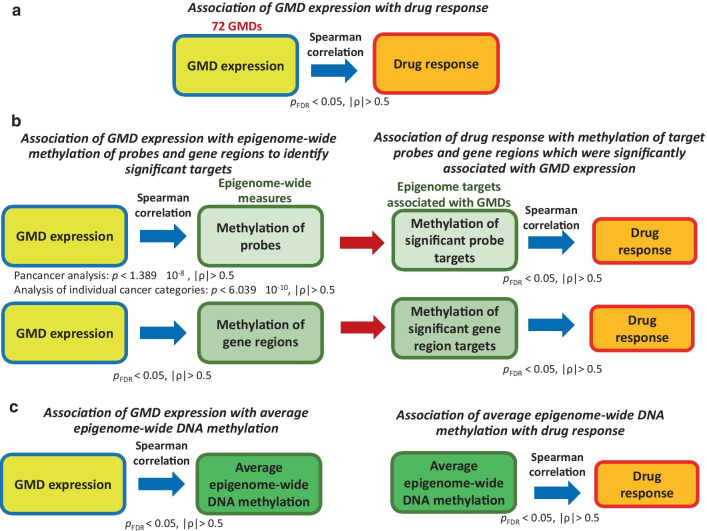
Overall design of the study. **a** Analysis of direct associations between GMD expression and drug response. **b** A two-step approach to identify possible indirect GMD effects on drug response. Epigenetic targets significantly associated with GMD expression were identified first, and then, the correlation of methylation of these significant targets with drug response was analyzed. **c**. Analysis of average DNA methylation values. In each analysis, we examined associations in all cancer categories combined (pancancer analysis) and in individual cancer types with ≥ 10 cell lines. GMD expression data included RNA-seq RPKM values for 72 GMDs. Epigenome-wide DNA methylation data included beta-values for 424,840 individual probes and gene region-averaged values for 93,591gene regions from 20,643 genes and noncoding RNA. Average epigenome-wide methylation values were computed as a mean of beta-values for 424,840 probes which passed the QC and filtering. Drug response measures consisted of 275 log(IC50) values for 255 anticancer agents obtained from CCLE and GDSC datasets. The criteria for identifying significant strong associations are provided below each diagram. *p*_FDR_, *p*-value adjusted for false discovery rate according to Benjamini–Hochberg procedure

To examine possible direct influences of GMD expression on drug response (Fig. 1), we analyzed Spearman correlation between RPKM expression measures of 72 GMDs listed in Additional file 1: Table S1 and log(IC50) of 255 antitumor agents. Significance of the associations was evaluated using the Benjamini–Hochberg adjustment procedure for false discovery rate (FDR) [17], while accounting for 255 agents and 72 genes. We identified the associations between GMD expression and log(IC50) which were both statistically significant (satisfying FDR adjusted *p* < 0.05) and strong (satisfying the absolute value of Spearman correlation coefficient |*ρ*| > 0.5) (Fig. 2a). Here and below, we refer to the FDR adjusted *p-*values as *p*_FDR_. We discuss the strength of statistically significant associations based on the absolute value of their Spearman correlation coefficient |*ρ*|.

All association analyses were performed in the combined dataset of different cancer categories (pancancer analysis including 645 cell lines), and also separately within each of the 23 individual cancer categories with at least 10 cell lines that had both methylation and expression data (Additional file 2: Table S2). The initial information about tumor sites was obtained from GDSC, CCLE, and Cellosaurus [67–69, 71, 72, 75, 76]. While many cancer categories used in our analysis were based on the Cancer Genome Atlas (TCGA) definitions, some cancer types from the same organ were grouped into broader categories in order to allow for an inclusion of a broader range of similar cell lines than those defined by TCGA. Several additional categories not presented in TCGA (e.g., small cell lung cancer, neuroblastoma, and others) were also analyzed (Additional file 2: Table S2). In the analysis stratified by individual cancer categories with ≥ 10 cell lines, we accounted for 23 cancer types in the FDR adjustment.

We also explored potential indirect mechanisms which may mediate the associations between GMD expression and drug response. We used Spearman correlation to identify the strongest significant associations between expression of the 72 GMDs and methylation of their epigenome targets by using methylation beta-values of 424,840 individual probes and 93,591 gene region methylation values averaged among the probes within each region (Fig. 2b). Among individual probes, in the combined pancancer analysis of all tumor types we searched for associations between GMD expression and methylation beta values with *p* < $\frac{{10}^{-6}}{72}$, i.e., *p* < 1.389 × 10^–8^, based on published recommendations [88] for the *p-*value threshold that would be appropriate for finding single gene associations with methylation probes of the HumanMethylation 450 K BeadChip array. We adjusted it by the number of GMDs for which the associations were examined. When analyzing associations between GMD expression and individual probes within each of the 23 cancer categories, we further adjusted this threshold by using *p* < $\frac{{1.389 10}^{-8}}{23}$  = 6.039 × 10^–10^. In the pancancer correlation analysis between GMD expression and gene region methylation, we used the FDR adjustment that accounted for 72 GMDs and 93,591 gene regions. In correlation analyses between GMD expression and gene region methylation stratified by cancer types, we also accounted for the 23 cancer types. In addition to using the *p-*value threshold, we focused on the strongest correlations that had the absolute value of Spearman |*ρ*| > 0.5. We made a distinction between the *cis-*correlations of expression of a GMD with methylation of its own probes (which could suggest the regulation of expression of that GMD by its methylation, or a possible copy number variation of that GMD which may affect both its methylation and expression measures) and the *trans-*correlations of each GMD with the probes located in other genes, according to the UCSC annotation of the HumanMethylation 450 K BeadChip array.

After identifying putative epigenome targets that were strongly and significantly correlated with GMD expression (Fig. 2b), we examined associations of DNA methylation of these epigenome targets with drug response. Spearman correlation analysis of methylation measures with log(IC50) of each of the 255 agents was performed for methylation beta-values of the target methylation probes and gene regions that had been strongly and significantly associated with GMD expression. Significance of correlation of methylation of individual target probes or gene regions with GMD expression and with log(IC50) was evaluated using the FDR adjustment, accounting for 255 agents and the number of target methylation probes and gene regions. Analysis within cancer categories also accounted for 23 cancer types. We focused on the strongest significant correlations with |*ρ*| > 0.5. If the number of such correlations was small, we provided an additional discussion of more modest significant correlations with |*ρ*| > 0.4.

In addition to the analysis of individual probes and gene regions, we also examined the association of GMD expression with epigenome-averaged DNA methylation and of epigenome-averaged DNA methylation with log(IC50) of antitumor agents (Fig. 2c). Epigenome-wide averaged DNA methylation was computed as a mean of beta-values among 424,840 methylation probes which passed the quality control (QC) and probe filtering. The resulting *p-*values were FDR adjusted for multiple testing. Separate analyses of average epigenome-wide DNA methylation were performed in the pancancer data and within 23 individual cancer categories with ≥ 10 cell lines.

Analyses were performed using Python v. 2.7.15, R v. 3.5.3, and rpy2 v. 2.8.5.

### Regression analysis of associations of cell line response to trametinib

Among the agents which were associated with GMD expression or with methylation status of epigenome targets in our study, sensitivity and resistance to the MEK inhibitor trametinib have been previously associated with specific DNA and protein sequence changes including BRAF V600E and KRAS or NRAS protein-changing variants [89, 90]. For those GMDs and target gene regions and probes which were significantly and strongly (|*ρ*| > 0.5) associated with response to trametinib either in the pancancer dataset or in any individual cancer category, we performed a regression analysis conditional on the presence of BRAF V600E or any non-synonymous *KRAS* or *NRAS* variant as predictors of trametinib response. We examined whether the association of GMD expression or methylation of their epigenome targets with response to trametinib remained statistically significant after accounting for the gene sequence variants known to affect sensitivity or resistance to trametinib. Information about the sequence variants in *BRAF, KRAS,* and *NRAS* was obtained from GDSC whole exome sequencing data [75]. Regression analysis was performed using the Imtest R package v. 0.9–36 for testing linear regression models, using log(IC50) as a dependent variable, and gene mutation status and GMD expression or probe or gene region methylation as predictor variables. The *p-*values for association of response to trametinib with GMD expression or with target probe and gene region methylation were FDR adjusted for multiple testing.

### Validation of the top study findings in publicly available independent datasets

In order to validate the top results from our correlation analyses between GMD expression, epigenome target methylation, and drug response, we used publicly available comprehensive independent datasets containing drug response, DNA methylation, and gene expression measures. Our first validation analysis used the NCI-60 cancer cell line panel dataset, previously screened by the National Cancer Institute, which we analyzed using CellminerCDB v. 1.2 [48, 91–93]. In the CellminerCDB analysis of NCI-60 cell line panel data, we examined Pearson correlation between GMD expression (measured as log2 of averaged gene expression measures from five microarray platforms, Affymetrix Human Genome HG-U95, Affymetrix Human Genome HG-U133, Affymetrix Human Genome U133 Plus 2.0, Affymetrix GeneChip Human Exon 1.0 ST, and Agilent Whole Human Genome Oligo arrays) and log (GI50) measures of drug response (representing drug activity measures in CellminerCDB multiplied by -1, in order to make the correlations in the CCLE-GDSC and NCI-60 datasets directly comparable) [48, 94]. We also analyzed NCI-60 data using CellminerCDB in order to validate significant correlations of GMD expression with target DNA methylation in the pancancer data. Because CellminerCDB utilizes gene level DNA methylation values which are inferred from probes located predominantly in the upstream gene regions [95], we used CellminerCDB NCI-60 DNA methylation data to confirm significant CCLE-GDSC associations of DNA methylation of upstream gene regions (TSS1500, TSS200, 5′UTR, and the 1st exon). CellminerCDB employs Pearson correlation in its analyses.

The second validation analysis used the NCI SCLC cell line dataset, containing measures for 66 small cell line cancer cell lines [86, 96]. It is available from the NCI Small Cell Lung Cancer Project site [97], with SCLC DNA methylation and transcript expression data also available from NCBI GEO (accession numbers GSE145156 and GSE73160). In the validation analysis using the NCI SCLC cell line data, we used Pearson correlation to examine associations of GMD expression, measured using Affymetrix GeneChip®Human Exon 1.0 ST Array, with log(IC50) measures of drug response, and Spearman correlation to analyze associations between DNA methylation of individual probes, measured using Illumina Infinium MethylationEPIC BeadChip, and average methylation of gene regions, with GMD expression and with log(IC50) measures of drug response, using methodology described in our earlier report [86]. Measures of miRNA methylation were not included in the validation analysis of SCLC data.

For additional validation of the epigenome targets identified in our analysis of CCLE-GDSC data, we explored the clinical relevance of the findings in pancreatic ductal adenocarcinoma (PAAD) based on the literature reports which analyzed patient survival data in TCGA and in other patient datasets.

### Searchable online resource

Our analysis generated an extensive set of tables with detailed information about the associations of genes affecting DNA methylation or demethylation. In order to provide the scientific community with the opportunity to independently explore these associations, we developed a web resource with dynamic searching and filtering features. The web resource is available at https://brb.nci.nih.gov/gmdtables/. It was developed using HTML, CSS, and the DataTables Javascript plug-in as highly flexible tools that allow researchers to visualize, search, filter, and download our results data for their own use. The online site also provides information about the 645 cancer cell lines used in our analysis.

## Results

### Association of GMD expression with drug response

Table 1 summarizes significant associations of GMD expression with log(IC50) which satisfied Spearman |*ρ*| > 0.4 and *p*_FDR_ < 0.05. Seven negative and positive correlations in individual cancer categories satisfied *p*_FDR_ < 0.05. All of them were strong (0.5171 ≤|*ρ*|≤ 0.7900; Table 1). The highest number (4) of significant associations was observed in breast cancer.

**Table 1:** Significant correlations of GMD expression with drug response in the pancancer dataset and in individual cancer categories satisfying |*ρ*| > 0.4 and *p*_FDR_ < 0.05

Cancer category	GMD	Agent	*ρ*	*p*_FDR_	Sample size
Pancancer	*KDM2B*	XMD13-2	− 0.4319	3.99 × 10^–24^	590
Pancancer	*KDM2B*	BMS-345541	− 0.4313	3.99 × 10^–24^	590
Pancancer	*KDM2B*	T0901317	− 0.4231	3.83 × 10^–23^	586
Pancancer	*DNMT1*	Zibotentan	− 0.4214	3.83 × 10^–23^	591
Pancancer	*KDM2B*	NPK76-II-72–1	− 0.4174	1.01 × 10^–22^	591
Pancancer	*APOBEC3G*	Z-LLNle-CHO	− 0.4148	2.64 × 10^–9^	225
Pancancer	*KDM2B*	Zibotentan	− 0.4139	2.37 × 10^–22^	591
Pancancer	*KDM2B*	Quizartinib	− 0.4095	8.98 × 10^–22^	589
Pancancer	*KDM2B*	UNC1215	− 0.4062	5.08 × 10^–21^	574
Pancancer	*KDM2B*	Daporinad	− 0.4057	7.77 × 10^–21^	569
Pancancer	*KDM2B*	Vorinostat	− 0.4057	2.13 × 10^–19^	527
Pancancer	*DNMT1*	XMD13-2	− 0.4043	2.87 × 10^–21^	590
Pancancer	*EHMT2*	NPK76-II-72–1	− 0.4041	2.87 × 10^–21^	591
Pancancer	*DNMT1*	Daporinad	− 0.4036	1.21 × 10^–20^	569
Pancancer	*KDM2B*	XMD14-99	− 0.4035	3.24 × 10^–21^	590
Pancancer	*KDM2B*	BX-912	− 0.4031	3.37 × 10^–21^	590
Pancancer	*KDM2B*	I-BET-762	− 0.4020	5.08 × 10^–21^	587
Pancancer	*KDM2B*	Tubastatin A	− 0.4011	5.97 × 10^–21^	587
BREAST	*GADD45A*	Refametinib	− 0.8026	0.0002	40
MATBCL	*BMI1*	5-Fluorouracil	− 0.7900	0.0440	27
SCLC	*APOBEC3A*	GSK1070916	0.7764	0.0399	29
BREAST	*APOBEC3C*	Cetuximab	− 0.7278	0.0242	38
BREAST	*APOBEC3G*	Cetuximab	− 0.7105	0.0399	38
BREAST	*GADD45A*	Trametinib	− 0.7012	0.0399	39
NSCLC	*IDH1*	(5Z)-7-Oxozeaenol	0.5171	0.0242	91

All agents listed in table were from the GDSC dataset. Abbreviations for cancer categories are provided in Table S2 and in the list of abbreviations. *ρ*, Spearman correlation coefficient; *p*_FDR_, FDR adjusted *p-*value; Sample size, the number of available cell lines in each category with available RNA-seq expression data and drug response data

**Fig. 3:**
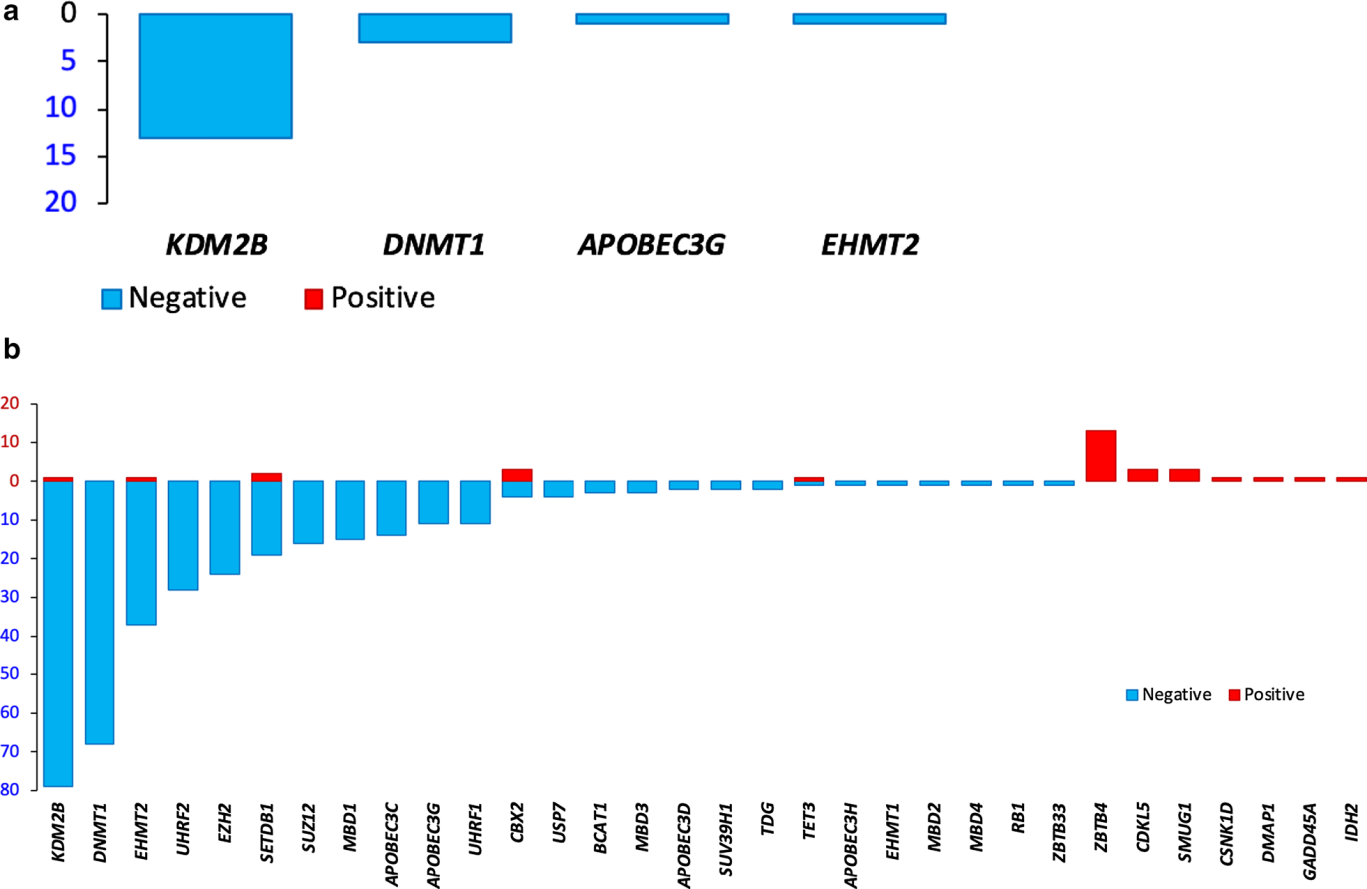
Numbers of significant correlations of GMD expression with log(IC50) measures of drug response satisfying *p*_FDR_ < 0.05. Positive correlations are shown as red bars directed upward, whereas negative correlations are shown as blue bars directed downward. A. Correlations satisfying Spearman |*ρ*| > 0.4. B. Correlations satisfying Spearman |*ρ*| > 0.3

Pancancer correlations were highly significant but did not reach |*ρ*| > 0.5. Four genes had modest correlations with |*ρ*| > 0.4 (Fig. 3a), all of which were negative, indicating that increased GMD expression was associated with drug sensitivity. They were *KDM2B* (13 correlations), *DNMT1* (3), *APOBEC3G* (1), and *EHMT2* (1). Additional file 3: Table S3 and Fig. 3b provide an expanded list of 379 significant pancancer correlations satisfying a relaxed threshold of |*ρ*| > 0.3 and *p*_FDR_ < 0.05. In the majority of them (91.8%, or 348 out of 379 correlations), increased GMD expression was associated with sensitivity. Expression of many GMDs, e.g., *KDM2B, DNMT1, EZH2, SETDB1, SUZ12, SUV39H1, EHMT1, EHMT2, BCAT1, MBD1, MBD2, MBD3, UHRF1, UHRF2, USP7, TDG, APOBEC3C, APOBEC3D, APOBEC3G,* and *APOBEC3H,* was predominantly associated with drug sensitivity (Fig. 3; Additional file 3: Table S3). In contrast, *ZBTB4, SMUG1,* and *CDKL5* expression was predominantly associated with drug resistance.

Many epigenetic drugs were associated with GMD expression. Increased expression of the histone demethylase *KDM2B* gene was associated with sensitivity to the HDACi vorinostat, tubastatin A, panobinostat, belinostat, CAY10603, VNLG/124, and AR-42, the bromodomain inhibitor I-BET-762, the SIRT1 inhibitor selisistat, the EHMT1/EHMT2 inhibitor UNC0638, and the DOT1L protein methyltransferase inhibitor SGC0946 (Table 1; Additional file 3: Table S3). Correlations of *KDM2B* expression with agents targeting histone modifications likely involve the epigenetic  role of KDM2B and its role in gene regulation [98].

Expression of the maintenance DNA methyltransferase *DNMT1* gene was associated with many epigenetic agents, including the HDACi tubastatin A, belinostat, VNLG/124, CAY10603, CUDC-101, and AR-42, the SIRT1 inhibitor selisistat, the EHMT1/EHMT2 inhibitor UNC0638, and the DOT1L inhibitor SGC0946 (Table 1; Additional file 3: Table S3). Their effects on DNMT1 may directly influence DNA methylation. For example, HDACi downregulate gene expression and protein levels of DNMT1, decrease its interaction with PRC2, and induce global DNA hypomethylation [50–52, 54, 55], and DNMT1 is a deacetylation target of SIRT1, which is inhibited by selisistat [29].

Expression of the H3K27 histone methyltransferase *EZH2* gene was modestly (-0.4 ≤ *ρ* < -0.3; Additional file 3: Table S3) associated with sensitivity to the HDACi vorinostat, tubastatin A, belinostat, the bromodomain inhibitor I-BET-762, and the DOT1L inhibitor SGC0946. Similar to *KDM2B* and *DNMT1*, *EZH2* associations with epigenetic drugs may involve direct interactions. EZH2, an important regulator of cancer gene expression [99], interacts with class I HDACs [100], and its protein levels and interaction with DNMT1 are downregulated by HDAC inhibitors [50, 55]. The weak but significant (*ρ* = -0.3028, *p*_FDR_ = 4.59 × 10^–12^) association of *EZH2* expression with sensitivity to I-BET-762 is surprising, as earlier reports found that EZH2 loss increased tumor sensitivity to bromodomain inhibitors [101, 102].

We observed many associations of sensitivity to HDACi and the bromodomain inhibitor I-BET-762 with elevated expression of a number of GMDs, e.g., *SETDB1, EHMT2, SUZ12, MBD1, UHRF2,* and *TDG* (Additional file 3: Table S3)*. MBD1* expression was associated with sensitivity to the EHMT1/EHMT2 inhibitor UNC0638. In contrast, *ZBTB4* and *GADD45A* expression was associated with resistance to HDACi. Associations with pretreatment expression of multiple GMDs are in agreement with the multifaceted actions of epigenetic agents which affect multiple molecular components [1, 100, 103].

In addition to epigenetic drugs, many GMDs were associated with other categories of antitumor agents (Table 1; Additional file 3: Table S3). Some correlations are directly related to their mechanisms of action. For example, *RB1* expression was correlated with sensitivity to palbociclib, a cyclin-dependent kinase (CDK) 4/6 inhibitor (*ρ* = -0.3060, *p*_FDR_ = 8.60 × 10^–11^; Additional file 3: Table S3), in agreement with sensitivity of Rb-positive cells to CDK 4/6 inhibitors, which target the cyclin D–CDK 4/6–Rb pathway, and with reduced *RB1* expression in cell lines resistant to palbociclib [104–106].

Other associations suggest indirect involvement of the epigenetic pathways in drug response. Elevated expression of *KDM2B, DNMT1, EHMT2,* and *UHRF1* was associated with sensitivity to daporinad, a nicotinamide phosphoribosyltransferase inhibitor (Table 1; Additional file 3: Table S3). *KDM2B, DNMT1, EZH2, UHRF1,* and *MBD1* were associated with sensitivity to the endotelin receptor A inhibitor zibotentan. Expression of *KDM2B, DNMT1, EHMT2, EZH2, SUZ12, MBD1, UHRF2,* and *SETDB1* was associated with sensitivity, and that of *ZBTB4* with resistance to the RIPK inhibitor XMD13-2 (Table 1; Additional file 3: Table S3). As sample sizes in individual tumor types were modest (Additional file 2: Table S2), many associations were significant in the pancancer analysis only. Their strength could be influenced by the differences in GMD expression and drug response among cancer types. Pancancer associations may indicate the GMD importance in response to the agents with similar activity across different tumor types.

### Association of GMD expression with DNA methylation of epigenome targets

In order to examine indirect modulation of drug response by GMDs via their influence on DNA methylation, we identified their genome methylation targets which were strongly and significantly associated with their expression (Fig. 2b).

#### Pancancer analysis of individual target probes and gene regions

Additional files 4, 5: Tables S4 and S5 provide the lists of strong significant pancancer associations of expression of 72 GMDs with DNA methylation. Analysis of 424,840 probes identified 1,905 strong GMD-probe correlations with *p* < 1.389 × 10^–8^ and |*ρ*| > 0.5 (Additional file 4: Table S4). They included 1770 highly significant GMD-probe *trans*-correlations involving target probes in other genes (0.5 < |*ρ*|≤ 0.7281, 1.57 × 10^–107^ ≤ *p* ≤ 3.75 × 10^–42^), which included 19 GMDs and 1,095 probes in 595 target genes. Analysis of gene regions identified 249 strong and significant correlations with GMD expression (*p*_FDR_ < 0.05, |*ρ*| > 0.5), including 236 *trans-*correlations, which involved 17 GMDs and 130 target genes (0.5 < |*ρ*|≤ 0.6719, 1.27 × 10^–79^ ≤ *p*_FDR_ ≤ 1.08 × 10^–37^; Additional file 5: Table S5).

**Fig. 4:**
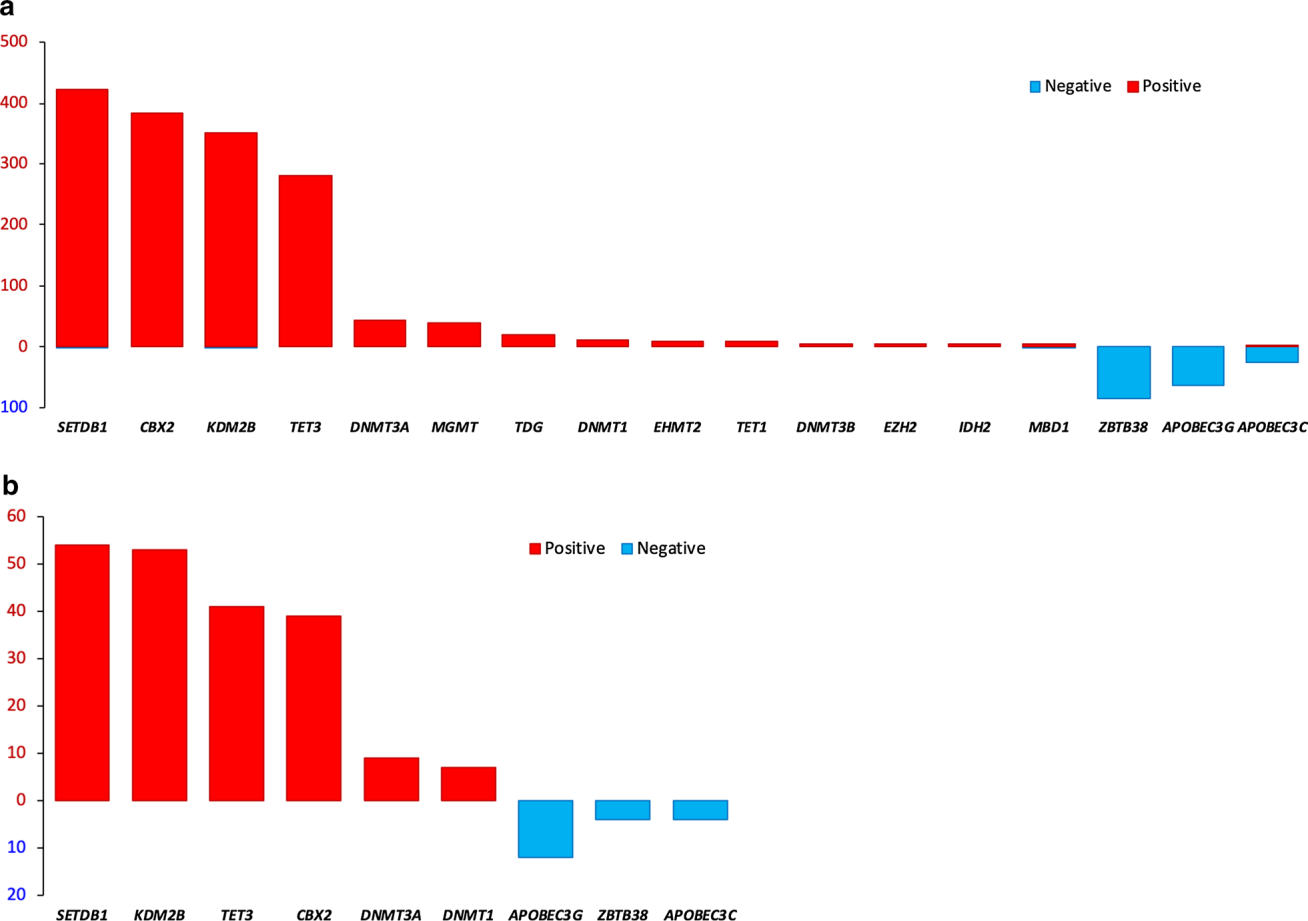
Graphical overview of the highest numbers of strong *trans-*correlations between GMD expression and methylation levels of target individual probes and gene regions in pancancer analysis. Shown are the counts of *trans-*correlations for GMDs presented in Table 2. Positive correlations are shown as red bars directed upward, whereas negative correlations are shown as blue bars directed downward. A. GMDs with *trans-*correlations with ≥ 5 probes which satisfied *p* < 1.389 × 10^–8^ and Spearman |*ρ*| > 0.5. B. GMDs with *trans-*correlations with ≥ 4 gene regions which satisfied *p*_FDR_ < 0.05 and Spearman |*ρ*| > 0.5

Among *trans-*correlations, expression of *BCAT1, CBX1, CBX2, DNMT1, DNMT3A, DNMT3B, EHMT1, EHMT2, EZH2, IDH2, KDM2B, MGMT, SETDB1, TDG, TET1,* and *TET3* was nearly exclusively positively associated with methylation of probes or gene regions (Table 2; Fig. 4; Additional files 6, 7: Tables S6 and S7). Expression of *APOBEC3C, IDH1,* and *ZBTB38* was exclusively, and that of *APOBEC3G* was predominantly negatively strongly associated with DNA methylation of other genes (Table 2; Fig. 4; Additional files 6, 7: Tables S6 and S7). *MBD1* was involved in a small number of both positive and negative correlations (Additional files 6, 7: Tables S6 and S7).

**Table 2:** GMDs with the highest numbers of strong *trans-*correlations between their expression and methylation levels of target individual probes and gene regions in pancancer analysis

GMD	Positive correlations	Negative correlations
*GMDs correlated with* ≥ *5 individual probes*		
*SETDB1*	422	1
*CBX2*	383	0
*KDM2B*	352	1
*TET3*	280	0
*DNMT3A*	43	0
*MGMT*	39	0
*TDG*	20	0
*DNMT1*	11	0
*EHMT2*	10	0
*TET1*	9	0
*DNMT3B*	6	0
*EZH2*	5	0
*IDH2*	5	0
*MBD1*	4	1
*ZBTB38*	0	86
*APOBEC3G*	0	63
*APOBEC3C*	2	24
*GMDs correlated with* ≥ *4 gene regions*		
*SETDB1*	54	0
*KDM2B*	53	0
*TET3*	41	0
*CBX2*	39	0
*DNMT3A*	9	0
*DNMT1*	7	0
*APOBEC3G*	0	12
*ZBTB38*	0	4
*APOBEC3C*	0	4

Listed are the counts of correlations satisfying *p* < 1.389 × 10^–8^ and Spearman |*ρ*| > 0.5 for individual probes and *p*_FDR_ < 0.05 and Spearman |*ρ*| > 0.5 for gene regions

Among GMDs involved in *trans*-correlations with ≥ 4 probes and ≥ 5 gene regions (Table 2; Fig. 4), *SETDB1, CBX2, KDM2B,* and *TET3* each had many positive associations with 280—422 probes and 39—54 gene regions. *MGMT, TDG, EHMT2, TET1, EZH2, IDH2,* and *MBD1* each had positive correlations with ≥ 4 probes (Table 2; Fig. 4). *ZBTB38* and *APOBEC3G* were involved only in negative *trans-*correlations with 86 and 63 probes, respectively, and with 4 regions each. *APOBEC3C* was predominantly involved in negative *trans-*correlations (Table 2; Fig. 4). All counts of the *cis-* and *trans-*correlations of GMD expression with probes and regions are listed in Additional files 6, 7: Tables S6 and S7.

Expression of all DNA methyltransferase genes had positive *trans*-correlations with DNA methylation. *DNMT3A* was strongly and significantly associated with 43 probes and 9 regions, *DNMT1* with 9 probes and 7 regions, and *DNMT3B* with 6 probes (Table 2; Fig. 4; Additional files 4, 5, 6, 7: Tables S4–S7), consistent with their functional roles. Interestingly, expression of hydroxymethylating genes was also positively associated with probe methylation, with 280 positive correlations for *TET1* and 9 for *TET3*. While their products are involved in oxidation of 5-mC to 5-hmC, which subsequently leads to DNA demethylation [13, 107], there have been both reports of the epigenome-wide TET effects on DNA hypomethylation and on increased methylation [108]. The DNA methylation microarray data used in our study did not distinguish between 5-mC and 5-hmC [88, 109], and positive associations of *TET3* and *TET1* (Table 2; Fig. 4; Additional files 4, 5, 6, 7: Tables S4–S7) likely involve measures of a mixture of 5-mC and 5-hmC.

GMD expression was associated with methylation of probes and regions in many important cancer genes (Additional files 4, 5: Tables S4 and S5). Selected examples of such associations are discussed in detail in Additional file 19: Data S1. For example, we observed epigenetic regulation of methylation of probes and/or gene regions of *ABL1, ABL2*, *MET, XRCC5, KIFC3,* and *TIMP.* Similarly, we observed associations of expression of multiple GMDs with a probe in *TGFBI,* whose product has been associated with poor prognosis in colorectal cancer and is a predictive biomarker for dasatinib sensitivity [110, 111]. Among the *ABC* family transporter genes, methylation of *ABCC1* and *ABCC3* was associated with GMD expression, suggesting multiple epigenetic pathways of their regulation.

Expression of multiple GMDs was associated with methylation of many probes and regions in genes involved in inflammation, e.g., *IRAK2* which encodes an activator of the NF-κB pathway, and tumor necrosis factor receptor genes *TNFRSF10B* and *TNFRSF1A.* Among the Hippo pathway components, methylation of *WWTR1* (*TAZ*) and *TEAD1* was significantly correlated with GMD expression (Additional files 4, 5: Tables S4 and S5; Additional file 19: Data S1).

GMD expression was correlated with methylation of other genes involved in epigenetic processes or global transcriptional regulation (Additional files 4, 5: Tables S4 and S5; Additional file 19: Data S1). Expression of the histone methyltransferase *SETDB1* and histone lysine demethylase *KDM2B* genes was positively correlated with methylation of the histone deacetylase *HDAC9,* suggesting *HDAC9* regulation by SETB1 and KDM2B or co-regulation among different histone modifiers. *KDM2B* and *TET3* expression was positively associated with methylation of *FTO,* which participates in RNA methylation [13]. *TET3* and *SETDB1* expression was positively correlated with methylation of *NNMT*, whose product promotes tumorigenesis and regulates the availability of methyl groups for cellular methylation reactions [112, 113]. *MED1* and *MED20* methylation was correlated with multiple GMDs, suggesting a potential influence of GMD expression on global transcriptional regulation. MED1 and MED20 are subunits of the mediator of RNA polymerase transcription. They participate in the Mediator complex, which is involved in transcriptional regulation of RNA polymerase II-dependent genes [114].

We also observed strong and highly significant *cis-*correlations of expression of 11 GMDs with their own probes and regions (Additional files 4, 5: Tables S4 and S5). Expression of *APOBEC3C, APOBEC3D, BCAT1, CBX2, DNMT3A, MGMT, IDH2, PHC2, TET1,* and *ZBTB38* had 106 positive and 29 negative *cis-*correlations with methylation of their own 135 probes (Additional file 4: Table S4). Expression of *APOBEC3B, APOBEC3C, APOBEC3D, BCAT1, MGMT,* and *PHC2* was associated with methylation of their gene regions (Additional file 5: Table S5)*.* The majority of *cis-*associations of the probe and region methylation of GMDs with their expression was strongly negative (Additional files 4, 5, 6, 7: Tables S4–S7), suggesting regulation of expression of these GMDs by their promoter methylation. Consistent with the well-documented repressive effect of the *MGMT* promoter methylation on its expression [37], methylation of the 5′ UTR, the 1st exon, and several individual probes of *MGMT* was negatively correlated with its expression.

#### Analysis of individual cancer categories

We observed strong and significant correlations of GMD expression with methylation of probes and gene regions in the stratified analysis among cancer categories. The use of the threshold of *p* < 6.039 × 10^–10^ for the probes identified 372 very strong correlations with 0.5801 ≤|*ρ*|≤ 1 (Additional file 8: Table S8), including 259 *trans-*correlations between 44 GMDs and probe methylation in 166 target genes. They represent the strongest and highly significant associations of GMD expression with individual probes. Methylation of many other probes was also correlated with GMD expression but did not satisfy the stringent *p-*value threshold (data not shown). Correlation analysis of GMD expression with methylation of gene regions identified 14,609 associations with 0.5 < |*ρ*|≤ 1 and *p*_FDR_ < 0.05, including 14,558 *trans-*correlations between expression of all 72 GMDs and the gene regions in 8,336 target genes (Additional file 9: Table S9).

Expression of many GMDs was correlated with methylation of multiple probes and gene regions (Table 3). A large number of associations was observed in chronic leukocytic leukemia (CLLE; Additional file 21: Fig. S2; Additional file 11: Table S11). Among the GMDs associated with ≥ 10 gene regions in CLLE, multiple positive correlations were observed for *UHRF1* (1320 positive associations), *CBX2* (687), *PHC2* (651), *CSNK1E* (536), *EHMT2* (392), *SUV39H2* (293), *IDH2* (165), *DNMT1* (154), *DNMT3B* (120), *EZH2* (106), *TRIM28* (56), *PCNA* (48), *SETDB1* (26), *UHRF2* (21), *DNMT3A* (19), *BMI1* (19), *MBD3* (16), and *SUV39H1* (14). Negative associations with ≥ 10 gene regions in CCLE cell lines were observed for *ZBTB38* (869), *EED* (47), *CDKL5* (33), *MBD2* (32), *APOBEC3A* (17), *AIDCDA* (15), and *APOBEC3B* (11). Consistent with their roles in DNA methylation and with the direction of associations in the pancancer dataset, *DNTM1, DNMT3A,* and *DNTM3B* expression was associated with increased methylation of many individual probes and gene regions in multiple tumor types (Table 3; Additional file 21: Fig. S2; Additional files 10, 11: Tables S10 and S11). Expression of *UHRF1,* whose product has multiple roles in DNA methylation including interactions with DNMT1, DNMT3a, DNMT3b and G9a, control of DNMT1 abundance, and targeting DNMT1 to hemimethylated DNA during replication (Additional file 1: Table S1) [24, 25, 32, 40, 115], was strongly associated with methylation of multiple probes and regions in many tumor types (Table 3; Additional file 21: Fig. S1; Additional files 10, 11: Tables S10 and S11). While *UHRF1* associations did not reach |*ρ*| > 0.4 in the pancancer data (Fig. 4; Additional files 6, 7: Tables S6 and S7), its expression had weaker positive significant associations with 12,611 probes with *p* < 1.389 × 10^–8^ and 0.3 < *ρ* < 0.4 and only 10 negative correlations with *p* < 1.389 × 10^–8^ and − 0.4 < *ρ* < − 0.3 (data not shown). This highlights the importance of URHF1 in DNA methylation in tumors.

**Table 3:** GMDs with the highest numbers of strong correlations between their expression and methylation levels of target probes and gene regions within individual cancer categories

Cancer type	GMD	Positive correlations	Negative correlations
*GMD correlations with* ≥ *5 individual probes*			
NSCLC	*MGMT*	25	0
CLLE	*UHRF1*	15	0
NSCLC	*DNMT3A*	12	0
NSCLC	*PHC2*	2	9
NSCLC	*CBX2*	8	2
BREAST	*EHMT1*	6	0
THCA	*CBX2*	6	0
CLLE	*SUV39H2*	5	0
COAD/READ	*APOBEC1*	0	34
CLLE	*ZBTB38*	0	25
BREAST	*SMUG1*	2	5
NSCLC	*SETDB1*	0	7
*GMD correlations with* > *100 gene regions*			
CLLE	*UHRF1*	1320	2
CLLE	*CBX2*	687	0
CLLE	*PHC2*	651	0
CLLE	*CSNK1E*	536	0
CLLE	*EHMT2*	392	0
COAD/READ	*SIRT1*	359	0
BREAST	*EHMT1*	268	39
CLLE	*SUV39H2*	293	1
SCLC	*DNMT1*	261	1
STAD	*TET1*	239	0
CLLE	*IDH2*	165	0
CLLE	*DNMT1*	154	0
SARCOMA	*IDH2*	139	5
STAD	*CBX1*	135	1
COAD/READ	*SUZ12*	126	1
CLLE	*DNMT3B*	120	0
GLIOMA	*TET3*	113	0
COAD/READ	*UHRF1*	111	0
CLLE	*EZH2*	106	0
BREAST	*BCAT1*	89	63
CLLE	*ZBTB38*	0	869
COAD/READ	*APOBEC1*	5	766
BREAST	*SMUG1*	34	273
BREAST	*TDG*	50	195
BREAST	*APOBEC3C*	22	97

Listed are the counts of correlations satisfying *p* < 6.039 × 10^–10^ and Spearman |*ρ*| > 0.5 for individual probes and *p*_FDR_ < 0.05 and Spearman |*ρ*| > 0.5 for gene regions

Associations of some GMDs were specific to individual cancer categories, suggesting heterogeneity of the mechanisms and of the strength of epigenetic interactions among cancer histologies. For example, in CLLE, *ZBTB38* expression was significantly (*p*_FDR_ < 0.05) negatively correlated with *ρ* < − 0.5 with 869 regions of other genes and had no positive *trans-*associations. By contrast, when using this threshold, *ZBTB38* had only 3 negative and 1 positive correlations with gene regions in NSCLC and only 1 negative correlation in breast cancer cell lines (Additional file 21: Fig. S2; Additional file 11: Table S11). It had no negative *trans-*correlations and 1 and 11 positive *trans-*correlations with gene regions in the COAD/READ and PAAD categories, respectively. Many other GMDs also had variable numbers of positive and negative strong associations in different tumors (Table 3; Additional file 21: Fig. S2; Additional files 8, 9, 10, 11: Tables S8–S11).

Similar to the pancancer analysis, we observed multiple strong significant associations of GMD expression with methylation of other GMDs and other genes involved in epigenetic processes and chromatin structure, maintenance, and regulation. For example, *APOBEC2* expression in bladder cancer was associated with methylation of the 5′ UTR and the 1st exon of the DNA demethylase *ALKBH2,* whose product removes N^1^-meA and N^3^-meC (*ρ* = − 0.8687; Additional files 1, 9: Tables S1 and S9) [38, 39].

We observed many strong tumor type-specific significant correlations of GMD expression with methylation of genes important in cancer. The detailed results are presented in Additional files 8, 9: Tables S8 and S9, and selected examples are discussed in Additional file 19: Data S1. They include associations of methylation of regions of the *ABL2* oncogene in breast cancer and in COAD/READ, and of the epidermal growth factor receptor *EGFR* gene in CLLE. Upstream gene regions of the tumor suppressor *RUNX1* were positively correlated with *DNMT3A* expression in liver hepatocellular carcinoma. *RUNX1* is downregulated in the early stages in hepatocellular carcinoma [116], and our findings suggest a potential role of DNA methylation in its regulation. The upstream region of *MYCN,* which may play a regulatory role in *MYCN* expression [86, 117], was associated with GMD expression in breast cancer and in COAD/READ. In CLLE, the body of *EGFR* was strongly positively correlated with *EHMT2* and *PHC2*.

In several tumor types, GMD expression was associated with methylation of the regions of *MLKL* and *RIPK3* encoding key players in necroptosis [118], *RIPK2* and *RIPK4,* which are involved in inflammatory signaling and NF-κB activation [119, 120], and *IRAK2, IRAK3,* and *IRAK4,* which mediate the toll-like receptor and interleukin-1 receptor signaling pathways and are involved in the NF-κB activation [121] (Additional file 9: Table S9; Additional file 19: Data S1). Numerous GMDs were strongly associated with methylation of components of the TNF-α signaling pathway [122] including *TNF* and other *TNF* family members, e.g., *TNSF11* (*RANKL*) and *TNSF13B* (*BAFF*) involved in activation of NF-κB signaling [123], *TNFAIP3* and *TNFAIP8L2* encoding TNF*-*α induced proteins, and the TNF receptor superfamily members.

GMD expression was strongly correlated with methylation of multiple components of the Hippo signaling pathway [122] (Additional files 8, 9: Tables 8 and S9; Additional file 19: Data S1), including *YAP1* in stomach adenocarcinoma (STAD), *WWTR1* (*TAZ*) in STAD, NSCLC, breast cancer, sarcoma, CLLE, and COAD/READ, *TEAD1* in CLLE, *TEAD2* in glioma and SCLC, *LATS1* in CLLE, *LATS2* in mature B-cell lymphoma, and *MST1* in CLLE. Methylation of *RASSF1, RASSF2, RASSF3, RASSF6, RASSF7,* and *RASSF9,* from the *RASSF* regulator family [122] was strongly associated with expression of several GMDs in a variety of cancer categories. As discussed above, *WWTR1* and *TEAD1* methylation was also associated with GMD expression in the pancancer data. These findings indicate a strong epigenetic regulation of the Hippo pathway.

We also found extensive epigenetic regulation of genome integrity. GMD expression was associated with methylation of *RAD51, RAD51C, RAD50, RAD1, RAD9A, RAD9B, RAD18, RAD21L1,* and *RAD23A* (Additional file 9: Table S9; Additional file 19: Data S1). RAD proteins are involved in DNA repair, chromosomal segregation, and checkpoint control [124–127]. We observed associations of methylation of *RAD51* in glioma, and of *RAD51C* and *RAD50* in CLLE. Upstream regions of *XRCC2, XRCC5,* and *XRCC6* were also positively associated with multiple GMDs. Methylation of the tumor suppressor gene *TP53BP1,* whose product mediates DNA damage response, was associated with GMD expression in COAD/READ, bladder cancer, and SCLC.

In several tumor categories, methylation of *TMEM173* (*STING*), *TREX1,* and *C6orf150* (*cGAS*) was correlated with GMD expression (Additional file 9: Table S9; Additional file 19: Data S1). Their products regulate the cytosolic DNA-sensing cGAS-STING innate immune pathway, activation of which is associated with improved tumor response to drug treatment and immunotherapy [128–134]. Our results suggest epigenetic influences on its regulation. Similarly to individual cancer types, we observed weaker significant (0.3001 < |*ρ*| ≤ 0.4124, 7.00 × 10^–28^ ≤ *p* ≤ 6.90 × 10^–15^) pancancer associations of upstream regions of these genes with multiple GMDs (data not shown).

### Association between methylation of target probes and gene regions and drug response

After identifying 1,306 target probes in 45 genes and 11,754 gene regions in 8,374 genes, which were strongly and significantly associated with GMD expression in pancancer analysis or in individual cancer types (Additional files 4, 5, 8, 9: Tables S4, S5, S8, and S9), we examined the association of their methylation with log(IC50) (Fig. 2). Only 4 probes and 3 regions had significant correlations with |*ρ*| > 0.5 in the pancancer data (*p*_FDR_ ≤ 2.94 × 10^–14^ for the probes and *p*_FDR_ ≤ 9.26 × 10^–17^ for the gene regions; Additional files 12, 13: Tables S12 and S13). The probe cg16411668 in a non-coding region was associated with *KDM2B*, *DNMT3A*, and *SETDB1* expression and with panobinostat sensitivity (*ρ* = -0.5245). The probes cg08422793 and cg20824939 in intergentic regions and cg20092122 in *BST2,* the bone marrow stromal antigen 2, were associated with sunitinib resistance (0.5027 ≤ *ρ* ≤ 0.5162) and with *APOBEC3G* and/or *APOBEC3C* expression. Consistent with the cg20092122 association, the TSS1500, TSS200, and the 1st exon of *BST2* were also associated with sunitinib resistance (*ρ* = 0.5167, *ρ* = 0.4622 and 0.4507; Additional file 13: Table S13). All these upstream regions were associated with *CBX2* expression. The 5′UTR of *SELPLG* was also associated with sunitinib resistance (*ρ* = 0.5305), and with *HDAC1* expression. The *CYR61* body was associated with panobinostat sensitivity (*ρ* = -0.5021) and with *KDM2B* and *SETDB* expression*.*

Using a less stringent cutoff of |*ρ*| > 0.4 for significant pancancer associations with log(IC50), we found 1,213 probe correlations and 714 gene region correlations (Additional files 12, 13: Tables S12 and S13) including many genes involved in cancer progression or drug transport. Examples of probe associations included *ABC* family transporters *ABCC1* and *ABCC3, SLC* transporters *SLC2A1, SLC4A7, SLC22A5, SLC25A22, SLC26A1, SLC39A11, SLC39A13,* and *SLC45A1,* the oncogenes *ABL1, ABL2, NF1,* and *RPTOR,* the *DUSP5* and *DUSP14* kinase genes*, RAD51L1* involved in homologous recombination repair [135], *FTO* and *HDAC9* encoding epigenetic factors, *IRAK2*, *MAP3K14*, *KIF3*, ubiquitin related genes *NEURL3* and *UBE2O,* and *NFIA* encoding the tumor-promoting transcription factor nuclear factor IA [136] (Additional file 12: Table S12). Associations of gene regions included the *ERBB2* (*HER2*) and *NOTCH3* oncogenes*,* the tumor suppressor *PHLDA1, CASP8* which plays a central role in apoptosis [118, 122], the N-myc interactor *NMI, PON2*, *CAMKK2*, *LIPG*, the *DUSP6* kinase gene, *KIF12*, the E-cadherin gene *CDH1,* the histone acetyltransferase *MYST1* (*KAT8*, or *MOF*), ubiquitin related *NDFIP2* and *UBA7* [137, 138], *NR1D2* (*Rev-erbβ*) encoding a transcriptional repressor, *RAP1GAP2*, *RASEF*, the glucocorticoid receptor gene *NR3C1*, *PPAP2C*, and the *SLC* transporter genes *SLC44A2* and *PQLC3* (*SCL66A3*) (Additional file 13: Table S13)*.*

We observed modest (|*ρ*| > 0.4) significant correlations involving both probes and the entire regions of many important genes (Additional files 12, 13: Tables S12 and S13). The 5′ UTR, 1st exon, and multiple probes in the tumor suppressor gene *DAPK3* were associated with the HDACi vorinostat and panobinostat. Panobinostat sensitivity was also correlated with methylation of a probe and the entire TSS1500 in *NNMT,* which controls the methylation potential of tumor cells [112], consistent with *NNMT* upregulation in a panobinostat resistant glioma cell line [139] and with the correlation of *NNMT* expression with vorinostat resistance [140]. Individual probes and the body of the oncogene *DDA1* were associated with sensitivity to the HDACi vorinostat and panobinostat, the bromodomain inhibitor I-BET-762, the PDK1 inhibitor BX-912, the LXR agonist T0901317, and the HER2 inhibitor TL-2–105 (Additional files 12, 13: Tables S12 and S13). The 5′UTR and its probes in *RUNX1* were associated with resistance to sunitinib, cyclopamine, and Z-LLNle-CHO. The 5′UTR, the body, and their probes in the transcriptional regulator *SP1* gene were associated with resistance to refametinib and tanespimycin. The 5′UTR and its probes in the transcriptional regulator *MAFK* gene were associated with sensitivity to the IKK inhibitor BMS-345541, the CRAF inhibitor TL-2-105, and the HDACi vorinostat. Many *MAFK* probes were also associated with other agents. The TSS200 of *TREX1* was associated with vorinostat sensitivity (*ρ* = -0.4101, *p*_FDR_ = 5.28 × 10^–19^), and the TSS200 of *TMEM173* (*STING*) was associated with sunitinib resistance (*ρ* = 0.4202, *p*_FDR_ = 5.09 × 10^–9^).

Methylation of many probes and regions was significantly associated with expression of *KDM2B, SETDB1, CBX2, EHMT1, DNTM1, DNMT3A, DNMT3B, TET1, TET2, TET3, MBD1, SMUG1, ZBTB38, APOBEC1, APOBEC3C, APOBEC3G,* and other GMDs, suggesting that GMDs may influence drug response via methylation of epigenome targets. Many probes correlating with drug response were associated with expression of multiple GMDs, suggesting intertwined pathways of epigenetic regulation.

Within tumor types, 904 probe-drug and 630 gene region-drug associations were strong and significant (|*ρ*| > 0.5, *p*_FDR_ < 0.05; Additional files 14, 15: Tables S14 and S15). Many of the same probes and regions also had weaker correlations with similar agents in the pancancer data (Additional files 12, 13: Tables S12 and S13). For example, cg25928474 in the *ABCC3* transporter gene had strong correlations with sensitivity to the HDACi AR-42 in ALL (*ρ* = -0.9636; Additional file 14: Table S14) and panobinostat in pancancer data (*ρ* = -0.4189; Additional file 12: Table S12). It had weaker (0.4 < |*ρ*| < 0.3) significant pancancer correlations with sensitivity to the HDACi vorinostat and the bromodomain inhibitor I-BET-762, and with other agents (data not shown). Its methylation was associated with expression of multiple GMDs including *TET3, TDG, SETDB1, ZBTB38, KDM2B,* and *CBX2*. Multiple probes and regions in other target genes, e.g., *ABL2, SP1, DAPK3, NF1, IRAK2, UBE2O,* and *FTO,* which were associated with drug response in the pancancer data, also had strong associations in individual tumor types (|*ρ*| > 0.5; Additional files 14, 15: Tables S14 and S15). Other examples of significant associations with log(IC50) in specific tumors included *WNT3A, WNT7A, FOXO3, FOX3P, WWTR1* (*TAZ*), *TEAD2, TNFRSF10B,* and *RASSF7*.

### Regression analysis of response to trametinib

After identifying significant methylation probes and gene regions associated with trametinib (Additional files 4, 5: Tables S4 and S5) and of the GMDs whose expression was associated with response to that agent with |*ρ*| > 0.5 (Table 1), we included them individually as predictor variables in multivariate regression analysis of trametinib response. We also used the mutation status of *BRAF, KRAS,* and *NRAS* as additional predictor variables. When BRAF V600E and non-synonymous changes in *KRAS* or *NRAS* were considered, methylation of the 5′UTR of *C7orf49* and of the probe cg00172872 in the intergenic region on 12q21.33 remained significantly associated with trametinib in pancancer and breast cancer (5.24 × 10^–13^ ≤ *p*_FDR_ ≤ 0.0023). BRAF V600E was also highly significant in these models both in breast cell lines and in the pancancer data (*p* ≤ 2.52 × 10^–5^), while the variants in *KRAS* or *NRAS* were significant in the pancancer data (*p* ≤ 2.23 × 10^–17^; data not shown). cg00172872 was associated with *CBX2, SETDB1,* and *TET3* expression (Additional files 4, 14: Tables S4 and S14), while the 5′UTR of *C7orf49* was associated with *GADD45A* (Additional files 9, 15: Tables S9 and S15). *GADD45A* expression was also strongly correlated with trametinib response in breast cancer (*ρ* = -0.7012; *p*_FDR_ = 0.0399; Table 1). When adding the BRAF V600E, *KRAS,* and *NRAS* mutation status to the model, association of *GADD45A* expression with trametinib in breast cell lines had *p* = 0.0003 prior to FDR adjustment, and *p*_FDR_ = 0.1566 after the adjustment (data not shown). These results suggest the importance of the *GADD45A* expression and *C7orf49* methylation in trametinib response. C7orf49 (CYREN) is a cell-cycle-specific inhibitor of classical non-homologous end joining of DNA double-strand break repair, regulating the selection of DNA double-strand repair pathway [141].

### Correlations of average epigenome methylation levels with GMD expression and drug response

Pancancer analysis showed very weak (|*ρ*| < 0.35, *p*_FDR_ < 0.05) significant correlations of expression of 43 GMDs with average epigenome methylation (data not shown). The strongest correlations were for *HELLS* (*ρ* = 0.3356, *p*_FDR_ = 1.41 × 10^–16^), *UHRF1, ZBTB38,* and *TET3* (*ρ* = 0.2423, -0.2951, and 0.2496, respectively; 4.82 × 10^–13^ ≤ *p*_FDR_ ≤ 2.37 × 10^–9^). *DNMT1, DNMT3A,* and *DNMT3B* had very weak positive correlations (*ρ* = 0.1902, 0.1886, and 0.2970, respectively; 6.63 × 10^–9^ ≤ *p*_FDR_ ≤ 7.29 × 10^–6^), consistent with their roles in promoting epigenome methylation (Fig. 4; Additional files 6, 7: Tables S6 and S7). Weak pancancer correlations are likely due to the differences in expression and methylation of individual genes among cancer categories.

Associations of epigenome methylation with GMD expression in individual tumor types satisfying *p*_FDR_ ≤ 0.15 are listed in Table 4. Many of them were very strong. Positive associations of *CSNK1E* in CLLE and *CBX2* in LAML reached significance (*ρ* = 0.8750 and 0.8018, *p*_FDR_ = 0.0306; Table 4), consistent with their positive associations with many gene targets in these leukemia types (Additional file 21: Fig. S2; Additional files 10, 11: Tables S10 and S11). CSNK1E, casein kinase 1ε, binds to DNMT1 and phosphorylates it, reducing its DNA-binding activity (Additional file 1: Table S1) [115, 142]. CBX2, a PRC1 member, is found in complexes with DNMT3A and DNMT3B [26, 143]. Positive correlations of *CBX1* in PAAD and of *SIRT1, SUZ12,* and *HELLS* in COAD/READ were nearly significant (0.5388 ≤ *ρ* ≤ 0.5506, 0.1886, and 0.2970; *p*_FDR_ = 0.0538; Table 4). *DNMT1, DNMT3A,* and *DNMT3B* associations were also positive but did not reach statistical significance, with the strongest correlations in CLLE (*ρ* = 0.7643, 0.5286, and 0.7750).

**Table 4:** Correlations of average epigenome methylation with GMD expression in individual cancer categories satisfying *p*_FDR_ < 0.15

Cancer category	GMD	Spearman *ρ*	*p*_FDR_	Sample size
CLLE	*CSNK1E*	0.8750	0.0306*	15
LAML	*CBX2*	0.8018	0.0306*	19
PAAD	*CBX1*	0.6606	0.0538	27
COAD/READ	*SIRT1*	0.5506	0.0538	43
COAD/READ	*SUZ12*	0.5471	0.0538	43
COAD/READ	*HELLS*	0.5388	0.0538	43
CLLE	*CBX2*	0.8036	0.0733	15
NSCLC	*UHRF1*	0.3563	0.0766	96
LAML	*MECP2*	0.7158	0.0845	19
PAAD	*EZH2*	0.6172	0.0845	27
GLIOMA	*TET3*	0.5439	0.0845	36
COAD/READ	*SUV39H2*	0.5115	0.0845	43
CLLE	*DNMT3B*	0.7750	0.0886	15
CLLE	*DNMT1*	0.7643	0.1029	15
LIHC	*CBX1*	0.7279	0.1029	17
GLIOMA	*PHC2*	− 0.5228	0.1119	36
CLLE	*UHRF1*	0.7393	0.1455	15
MATBCL	*UHRF2*	0.5291	0.1455	33
NSCLC	*MBD1*	− 0.3169	0.1455	96

An asterisk (*) indicates statistically significant associations with *p*_FDR_ < 0.05

Pancancer correlations of the average genome methylation with drug response were very weak, even though 14 agents reached significance (*p*_FDR_ < 0.05; data not shown). The majority of the correlations were weakly negative, suggesting a weak trend for higher sensitivity of more methylated cell lines. Only lapatinib, ZG-10, and WZ-1–84, had |*ρ*| > 0.2 (-0.2106 ≤ *ρ* ≤ -0.2064; *p*_FDR_ ≤ 0.0447; data not shown).

Stratified analysis within tumor types identified a strong and significant correlation between epigenome methylation in bladder cancer and sensitivity to the CDK inhibitor THZ-2–49 (*ρ* = -0.8596, *p*_FDR_ = 0.0243; data not shown). Two correlations in COAD/READ were strong with *p*_FDR_ < 0.15, including sensitivity to the PDK-1 inhibitor BX795 and the proteasome inhibitor MG-132 (*ρ* = -0.6376 and -0.8791, *p*_FDR_ = 0.126 for both; data not shown). The biological mechanisms of these associations require further investigation.

### Validation of the findings and their clinical significance using independent datasets

Among pancancer correlations of GMD expression with drug response presented in Table 1, which satisfied Spearman |*ρ*| > 0.4 and *p*_FDR_ < 0.05, seven associations had both GMD expression data and log(GI50) drug response data for the same agents available in the NCI-60 dataset in CellminerCDB (Additional file 16: Table S16). Among them, *KDM2B* expression was strongly and highly significantly correlated with sensitivity to the HDAC inhibitor vorinostat in the NCI-60 cancer cell lines (Pearson *r* = -0.51, *p* = 4.3 × 10^–5^), providing a strong support for our initial finding of this association in the CCLE-GDSC dataset. Four additional associations for *KDM2B, DNMT1,* and *APOBEC3G* had the same direction of association between GMD expression and drug sensitivity both in the CCLE-GDSC and NCI-60 datasets, but they did not reach statistical significance in the NCI-60 data (Additional file 16: Table S16).

Additional file 17: Table S17 shows the strength of Pearson correlation in the NCI-60 data from CellminerCDB, used for validation of significant correlations between GMD expression and DNA methylation of upstream gene regions (TSS1500, TSS200, 5′UTR, and the 1st exon) in the pancancer CCLE-GDSC data from Additional file 5: Table S5. Among the 116 significant correlations from CCLE-GDSC data listed in Additional file 17: Table S17 which also had comparable NCI-60 data (GMD expression and gene-averaged methylation derived from the upstream probes) in CellminerCDB, 63 (54% of the total) had both the Pearson correlation *p* < 0.05 in the NCI-60 data and the same direction of association in both datasets, confirming our initial findings. Many additional GMD-target gene associations in Additional file 17: Table S17 had the same direction of correlation both in the CCLE-GDSC and NCI-60 datasets but did not reach statistical significance.

We also observed a strong and consistent confirmation of our findings in an independent NCI SCLC dataset consisting of 66 small cell lung cancer cell lines, which we had generated previously [86]. Additional file 18: Table S18 provides Spearman correlation results between GMD expression and DNA methylation of gene regions in 66 SCLC cell lines. They validate the significant findings in the SCLC category of the CCLE-GDSC data from Additional file 9: Table S9 that had *p*_FDR_ < 0.05 and |*ρ*| > 0.5. Among 734 significant GMD-gene region correlations with available data in both datasets, 521 (71%) had both associations in the same direction and *p* < 0.05 in the independent NCI SCLC dataset. Among validated results for multiple associated GMDs in Additional file 18: Table S18, we note multiple correlations involving the *KMT2A* (*MLL*) gene which is frequently mutated in SCLC, and *EZH2*, an important epigenetic drug target in SCLC, pharmacologic inhibition of which suppresses SCLC growth and chemoresistance [144–146].

We were also able to validate several significant correlations of individual target epigenome probes and gene regions with drug response in the SCLC category in the CCLE-GDSC dataset (Additional files 14, 15: Tables S14 and S15) using the associations in the NCI SCLC dataset, even though these two datasets contained many different agents and used two different Illumina methylation arrays. We used the NCI SCLC dataset to confirm the associations of the TSS1500 of *CXCL17* and TSS1500 of PPR18 with response to docetaxel and of the probes cg0260189 in the body of *BIK* with docetaxel, cg04619882 in the body of *KIAAA1324* with dactolisib, and cg04619885 in the body of *UBE2O* with PD0325901 (0.2503 ≤|*ρ*|≤ 0.3623, 0.0029 ≤ *p* ≤ 0.0427, and the direction of associations was also identical in both datasets; data not shown). For some agents which were unique to the CCLE-GDSC screen, confirmation of clinically important associations with epigenomic targets may be suggested based on indirect evidence. For example, methylation of the TSS1500 of *TEAD2* in the SCLC category of the CCLE-GDSC dataset was associated with resistance to the mTOR inhibitor temsirolimus (Additional file 15: Table S15). It is consistent with an earlier report by an independent group using SCLC CCLE cell lines and with our previous findings in the NCI SCLC dataset, which showed that increased methylation and low expression of the genes encoding TEAD co-activators YAP1 and TAZ in the Hippo pathway in SCLC were associated with resistance to multiple mTOR inhibitors [86, 147, 148].

We further evaluated the available indirect support for the potential clinical significance of our findings in pancreatic adenocarcinoma, by examining published reports based on patient data. Among the five genes whose probes and/or region methylation was associated with in vitro drug response in our analysis of the PAAD category in the CCLE-GDSC dataset (Additional files 14, 15: Tables S14 and S15), *FMOD* had been previously reported to be associated with patient survival. It encodes fibromodulin, an extracellular matrix protein overexpressed in pancreatic ductal adenocarcinoma [149]. In our study, methylation of the *FMOD* gene body in PAAD was associated with response to the Hsp90 inhibitor 17-AAG (17-allylamino-17-demethoxygeldanamycin; Additional file 15: Table S15). FMOD protein expression had been previously associated with PAAD patient survival in the Queensland Centre for Medical Genomics dataset [149]. In other cancer categories, multiple studies have reported an association of upregulation of *FMOD* with poor patient survival in TCGA glioblastoma patients, and its product has been suggested to have an immunosuppressive role, whereas the silencing of *FMOD* leads to apoptosis in CCLE [150–152].

In our analysis of the CCLE-GDSC data, methylation of a probe and the gene body of *TPO* (encoding thyroid peroxidase) was associated with response to the c-Met and NPM-ALK inhibitor PF-2341066 in the PAAD category (Additional files 14, 15: Tables S14 and S15). *TPO* was previously reported to be among the most mutated genes in PAAD patient tumors in TCGA, suggesting a possible combined influence of epigenetic regulation of this gene and the mutational landscape on treatment response [153].

## Discussion

Using patient-derived cell line genomic and drug response data we identified significant associations of 72 important GMDs with drug response and with DNA methylation based on multiple probes across the epigenome. We were able to confirm many associations in independent datasets using direct validation of comparable associations in the NCI-60 and NCI SCLC cancer cell line panels, and indirect evidence from reports on PAAD patient data. Our results provide a resource for future studies of GMDs which may influence methylation of a particular gene of interest, or analyses to explore direct and indirect associations of GMDs with tumor cell line response for specific therapeutic and pharmacological agents. Expression of multiple GMDs was strongly and significantly correlated with response to a variety of agents, even though the associations in the pancancer data were modest. GMD expression had widespread associations with methylation of genes involved in tumor development and progression and in drug response, suggesting multiple overlapping regulatory influences on the epigenome.

When analyzing indirect GMD effects on drug response (Fig. 2B), we used the threshold of the Spearman correlation coefficient *ρ*, to focus on the strongest significant correlations of GMD expression with methylation of their targets, and on correlations of methylation of the most strongly associated targets with drug response. Individual GMDs also had multiple statistically significant weaker correlations with their targets which we did not report. For example, we identified 1,905 strong significant correlations of GMD expression with methylation of individual probes in the pancancer dataset satisfying *p* < 1.389 × 10^–8^ and Spearman |*ρ*| > 0.5 (Additional file 4: Table S4). When examining weaker GMD-probe correlations using the same significance threshold of *p* < 1.389 × 10^–8^, we found 24,904 associations with |*ρ*| > 0.4, and 254,827 correlations with |*ρ*| > 0.3 (data not shown). These results suggest common and complex influence of GMDs on DNA methylation in tumor cells. Weaker associations may indicate important biological influences of GMDs on cancer cell regulation, possibly under specific conditions or in subsets of tumor cells with specific mutational and/or expression profiles.

Many compounds, e.g., MS-275, JQ12, LAQ824, tubastatin A, VNLG/124, AR-42, CUDC-101, belinostat, CAY10603, vorinostat, panobinostat, UNC0638, SGC0946, JQ1, I-BET-762, and PFI-1, included HDAC inhibitors, inhibitors of histone methylation, and bromodomain inhibitors directly targeting epigenetic processes. Many of them directly target GMD products, e.g., HDAC1 is one of the targets of vorinostat, and EHMT1 and EHMT2 are targeted by UNC0638 [71].

While the associations of methylation of the target probes and gene regions with log(IC50) may suggest a possible regulation of drug sensitivity or resistance resulting from DNA methylation on gene expression, many correlations with methylation targets involved epigenetic agents, which may suggest additional epigenetic mechanisms. Examples include the HDACi panobinostat, vorinostat, and AR-42 and the bromodomain inhibitor I-BET-762 (Additional files 12, 13, 14, 15: Tables S12–S15). Examples of correlations of methylation of target genes with response to epigenetic drugs include methylation of *DAPK3, DDA1, NNMT, MAPK, TREX1,* and *ABCC3.* Even though methylation of those genes was measured prior to treatment, such genes may not necessarily directly affect sensitivity or resistance to epigenetic drugs. While a direct involvement of their products in the response to epigenetic agents is possible, another potential explanation could be that correlations involving methylation of specific target genes may indicate more global influences of different levels of GMD expression on epigenome methylation prior to treatment. In that case, methylation of specific target genes could be a marker of the overall epigenetic activity of one or more GMDs affecting multiple target genes, rather that indicate a direct influence of a specific target gene on drug response. Furthermore, in addition to their effect on DNA methylation, many GMDs analyzed in this study have other epigenetic or regulatory roles which are targeted by some of the agents. Some GMDs, e.g., HDACs, may indirectly regulate gene expression by modifying a diverse set of protein targets including transcription factors [154]. Further biological investigation may be needed to address whether the correlations of drug response with DNA methylation of target genes which were associated with GMD expression may be explained by the mechanisms involving the action of specific target gene products (e.g., by an effect of a transporter on a drug concentration within a cancer cell) or by broad non-specific effects of pretreatment GMD expression, which affects DNA methylation of multiple genes in the epigenome.

Our methylation dataset was restricted to the combined measurements of 5-mC and 5-hmC using the Illumina Infinium HumanMethylation450 BeadChip array. The products of several GMDs analyzed in this study, e.g., TET1, TET2, TET3, and TDG, generate 5-fC and 5-caC, whereas MGMT demethylates O^6^-meG, and the action of ALKBH2, and ALKBH3 results in the removal of N^1^-meA and N^3^-meC [1, 13, 37–39] (Additional file 1: Table S1). Drug resistance mechanisms involving some of these pathways, such as the role of *MGMT* expression in temozolomide resistance, were not detected in our study which used in vitro assay measures, likely because temozolomide is a prodrug which is converted to an active compound in the body, but possibly inconsistently in in vitro screening assays [155]. Similarly, we did not analyze methyladenine modifications as they were not captured in the available methylation data.

Altered GMD function in tumors can arise both from DNA mutations and transcriptional changes [4, 10, 144]. We analyzed the variation in GMD expression and did not examine GMD mutation status. As some GMDs may also have gain-of-function or loss-of-function variants in malignant cells, future large-scale analyses may investigate how drug response of tumor cells may be jointly influenced by DNA and protein sequence changes in GMDs, their copy number variation, gene fusions involving GMDs, and variation in GMD expression. Drug response may also be affected by the sensitivity or resistance mutations acquired by the genes encoding drug targets or by additional genes. Our regression analysis of trametinib response confirmed associations for *GADD45A* and its putative epigenome targets while accounting for the mutation status of *BRAF* V600E, *KRAS,* and *NRAS*.

Our study provides an extensive reference set of associations between expression of GMDs, their methylation of their epigenome targets, and response to drug treatment in a variety of cancer categories. These results provide a new insight into the epigenetic landscape of molecular interactions in tumors and suggest potential mechanisms of epigenetic influences on tumor cell response to a variety of chemotherapeutic agents.

## Conclusions

We identified multiple associations of GMD expression with drug response and with DNA methylation of individual probes and gene regions in the epigenome. Methylation of many epigenome targets was correlated with response to treatment. Our findings suggest potential direct and indirect influences of GMD expression on drug response, which may be mediated by interconnected regulation of DNA methylation pathways.

## Supplementary Information

**Additional file 1**: Table S1. Genes directly or indirectly involved in DNA methylation and demethylation which were included in analysis.

**Additional file 2**: Table S2. Numbers of cell lines with available CCLE and GDSC data, analyzed in each cancer category.

**Additional file 3**: Table S3. Significant correlations of GMD expression with drug response in the pancancer dataset satisfying |r| > 0.3 and *p*_FDR_ < 0.05.

**Additional file 4**: Table S4. Significant strong *trans-* and *cis-*correlations of GMD expression with methylation of individual probes in the pancancer dataset satisfying *p* < 1.389 × 10^–8^ and |*ρ*| > 0.5.

**Additional file 5**: Table S5. Significant strong *trans-* and *cis-*correlations of GMD expression with methylation of target gene regions in the pancancer dataset satisfying *p*_FDR_ < 0.05 and |*ρ*| > 0.5.

**Additional file 6**: Table S6. Numbers of *cis-* and *trans-*correlations of GMD expression with methylation of individual probes in the pancancer dataset satisfying *p* < 1.389 × 10^–8^ and |*ρ*| > 0.5.

**Additional file 7**: Table S7. Numbers of *cis-* and *trans-*correlations of GMD expression with methylation of gene regions in the pancancer dataset satisfying *p*_FDR_ < 0.05 and |*ρ*| > 0.5.

**Additional file 8**: Table S8. Epigenome-wide Spearman correlations of GMD expression with methylation of individual probes satisfying *p* < 6.039 × 10^–10^ and |*ρ*| > 0.5 in 23 individual cancer categories with at least 10 cell lines.

**Additional file 9**: Table S9. Epigenome-wide Spearman correlations of GMD expression with methylation of gene regions satisfying *p*_FDR_ < 0.05 and |*ρ*| > 0.5 in 23 individual cancer categories with at least 10 cell lines.

**Additional file 10**: Table S10. Numbers of *cis-* and *trans-*correlations of GMD expression with methylation of individual probes in the 23 individual cancer categories with at least 10 cell lines, satisfying *p* < 6.039 × 10^–10^ and |*ρ*| > 0.5.

**Additional file 11**: Table S11. Numbers of *cis-* and *trans-*correlations of GMD expression with methylation of gene regions in the 23 individual cancer categories with at least 10 cell lines, satisfying *p*_FDR_ < 0.05 and |*ρ*| > 0.5.

**Additional file 12**: Table S12. Significant correlations of methylation of target probes with drug response in the pancancer dataset satisfying *p*_FDR_ < 0.05 and a relaxed threshold of |*ρ*| > 0.4.

**Additional file 13**: Table S13. Significant correlations of methylation of target gene regions with drug response in the pancancer dataset satisfying *p*_FDR_ < 0.05 and a relaxed threshold of |r| > 0.4.

**Additional file 14**: Table S14. Significant correlations of methylation of target probes with drug response in the 23 individual cancer categories with at least 10 cell lines, satisfying *p*_FDR_ < 0.05 and |*ρ*| > 0.5.

**Additional file 15**: Table S15. Significant correlations of methylation of target gene regions with drug response in the 23 individual cancer categories with at least 10 cell lines, satisfying *p*_FDR_ < 0.05 and |r| > 0.5.

**Additional file 16**: Table S16. Significant correlations of GMD expression with drug response in the CCLE-GDSC pancancer dataset from Table 1 which had available data for association analysis in CellminerCDB.

**Additional file 17**: Table S17. Significant strong *trans*-correlations of GMD expression with methylation of upstream target gene regions in the CCLE-GDSC pancancer dataset from Additional file 5: Table S5 and their associations in the NCI-60 dataset in CellminerCDB.

**Additional file 18**: Table S18. Significant strong *trans*-correlations of GMD expression with methylation of upstream target gene regions in the SCLC category of the CCLE-GDSC dataset from Additional file 9: Table S9 and their associations in an independent SCLC dataset of 66 cancer cell lines.

**Additional file 19**: Data S1. Selected examples of association of GMD expression with DNA methylation of epigenome targets in individual cancer categories.

**Additional file 20**: Figure S1. Plots showing the distribution of DNA methylation values among 424,840 individual probes, the combined distribution of DNA methylation among 93,591 gene regions, and separate distribution for each gene region category in the 645 cell lines in the pancancer dataset.

**Additional file 21**: Figure S2. A graphical overview of the numbers of strong *trans-*correlations between GMD expression and methylation of gene regions in selected cancer categories, satisfying *p*_FDR_ < 0.05 and |*ρ*| > 0.5. Numbers of *trans*- and *cis-*correlations in these and additional cancer categories are provided in Additional file 11: Table S11. Positive correlations are shown as red bars directed upward, whereas negative correlations are shown as blue bars directed downward. (A) Chronic leukocytic leukemia (CLLE). (B) Colon adenocarcinoma and rectum adenocarcinoma (COAD/READ). (C) Breast cancer (BREAST).

## Data Availability

All CCLE, GDSC, and SCLC data used in this project are publicly available online. Information about their location is provided in the Methods section. The significant findings from our study, which are reported in Additional file 1, 3–15: Tables S1 and S2–S15, are available in a searchable format at https://brb.nci.nih.gov/gmdtables/.

## Abbreviations

5-caC: 5-Carboxylcytosine
5-fC: 5-Formylcytosine
2-HG: 2-Hydroxyglutarate
5-hmC: 5-Hydroxymethylcytosine
5-hmU: 5-Hydroxymethyluracil
5-mC: 5-Methylcytosine
17-AAG: 17-Allylamino-17-demethoxygeldanamycin
17-DMAG: 17-Dimethylaminoethylamino-17-demethoxygeldanamycin
AICDA (AID): Activation-induced cytidine deaminase
αKG: α-Ketoglutarate
ALL: Acute lymphocytic leukemia
AML (LAML): Acute myeloid leukemia
APOBEC1: Apolipoprotein B mRNA editing activity DNA deaminase 1
APOBEC2: Apolipoprotein B mRNA editing activity DNA deaminase 2
APOBEC3A: Apolipoprotein B mRNA editing activity DNA deaminase 3A
APOBEC3C: Apolipoprotein B mRNA editing activity DNA deaminase 3C
BER: Base excision repair
BLADDER: Bladder cancer
BREAST: Breast cancer
CCLE: Cancer Cell Line Encyclopedia
CESC: Cervical squamous cell carcinoma and endocervical adenocarcinoma
CAMKK2: Calcium/calmodulin-dependent protein kinase kinase 2
CLLE: Chronic lymphocytic leukemia
CDK: Cyclin-dependent kinase
COAD/READ: Colon adenocarcinoma and rectum adenocarcinoma
DA: Duodenal adenocarcinoma
DNMT: DNA methyltransferase
DNMT1: DNA methyltransferase 1
DNMT3A: DNA methyltransferase 3A
DNMT3B: DNA methyltransferase 3B
DNMT3L: DNA methyltransferase 3-like
EC: Esophageal cancer
EGFR: Epidermal growth factor receptor
EZH2: Enhancer of zeste homolog 2
FDR: False discovery rate
GADD45A: Growth arrest and DNA damage 45 protein A
GDSC: Genomics of Drug Sensitivity in Cancer
GEO: Gene Expression Omnibus
GLIOMA: Glioma brain tumors
GMD: Gene affecting DNA methylation or demethylation
H3K9: Histone H3 lysine 9
HDAC: Histone deacetylase
HDACi: Histone deacetylase inhibitors
HNSC: Head and neck squamous cell carcinoma
Hsp90: Heat shock protein 90
IDH: Isocitrate dehydrogenase
IDH1: Isocitrate dehydrogenase 1
IDH2: Isocitrate dehydrogenase 2
IRAK2: Interleukin-1 receptor-associated kinase 2
KIF3: Kinesin superfamily protein 3
LCML: Chronic myelogenous leukemia
LIHC: Liver hepatocellular carcinoma
LIPG: Endothelial lipase
MAP3K14: Mitogen-activated protein kinase kinase kinase 14
MATBCL: Mature B-cell lymphoma
MB: Medulloblastoma
MBD1: Methyl-CpG-binding domain protein 1
MBD2: Methyl-CpG-binding domain protein 2
MBD3: Methyl-CpG-binding domain protein 3
MBD4 (MED1): Methyl-CpG-binding domain protein 4
MEL: Melanoma
MESO: Mesothelioma
MeCP2: Methyl-CpG-binding protein 2
MGMT: O(6)-methylguanine-DNA methyltransferase
MISC: Other miscellaneous categories of cancer including rare cancers or cancers with unspecified information
MM: Multiple myeloma
N^1^-meA: 1-Methyladenine
N^3^-meC: 3-Methylcytosine
NCBI GEO: National Center for Biotechnology Information Gene Expression Omnibus
ncRNA: Non-coding RNA
NEURL3: Neutralized E3 ubiquitin protein ligase 3
NFIA: Nuclear factor I
NR1D2: Nuclear receptor subfamily 1 group D member 2
NSCLC: Non-small cell lung cancer
O^6^-meG: O^6^-methylguanine
OVARIAN: Ovarian cancer
PAAD: Pancreatic adenocarcinoma
Pancancer: Combined analysis of all cancer categories
PARP: Poly(ADP-ribose) polymerase
PCNA: Proliferating cell nuclear antigen
PNET: Primitive neuroectodermal tumors
PPAP2C: Phosphatidic acid phosphatase type 2C
PRA: Prostate adenocarcinoma
PRC: Polycomb repressive complex
PRC1: Polycomb repressive complex 1
PRC2: Polycomb repressive complex 2
QC: Quality control
RAP1GAP2: RAP1 GTPase activating protein 2
RASEF: The RAS and EF-hand domain containing protein
RCC: Renal cell carcinoma
RIPK: Receptor-interacting protein kinase
SAR: Sarcoma
SCLC: Small cell lung cancer
STAD: Stomach adenocarcinoma
STAT: Signal transducer and activator of transcription
TCGA: The Cancer Genome Atlas
TDG: Thymine-DNA glycosylase
TET: Ten-eleven translocation
TET1: Tet methylcytosine dioxygenase 1 (ten-eleven translocation-1)
TET2: Tet methylcytosine dioxygenase 2 (ten-eleven translocation-2)
TET3: Tet methylcytosine dioxygenase 3 (ten-eleven translocation-3)
THCA: Thyroid carcinoma
TNF: Tumor necrosis factor
UBE2O: (E3-independent) E2 ubiquitin-conjugating enzyme
UCEC: Uterine corpus endometrial carcinoma
USP7 (HAUSP): Herpes virus–associated ubiquitin-specific protease
