# Supplementary material for: Association of expression of epigenetic molecular factors with DNA methylation and sensitivity to chemotherapeutic agents in cancer cell lines
Source: Clin Epigenetics. 2021 Mar 6;13:49. doi: 10.1186/s13148-021-01026-4 (PMC7936435; doi:10.1186/s13148-021-01026-4)
Supplement: Supplementary file 1 — Additional file 1: Table S1. Genes directly or indirectly involved in DNA methylation and demethylation which were included in analysis. [file 13148_2021_1026_MOESM1_ESM.pdf]

**Table S1.** Genes directly or indirectly involved in DNA methylation and demethylation which were included in analysis

| **GMD** | **Synonyms** | **Full name** | **Mechanisms of effect on DNA methylation levels** | **References** |
|---|---|---|---|---|
| *DNMT1* | | DNA methyltransferase 1 | Maintenance DNA methylation | [1] |
| *DNMT3A* | | DNA methyltransferase 3A | *De novo* DNA methylation | [1] |
| *DNMT3B* | | DNA methyltransferase 3B | *De novo* DNA methylation | [1] |
| *DNMT3L* | | DNA methyltransferase 3-like protein | Stimulation of enzymatic activity of DNMT3A and DNMT3B, role in imprinting | [1-4] |
| *TET1* | | Tet methylcytosine dioxygenase 1 (ten-eleven translocation-1) | DNA demethylation via conversion of 5-mC to 5-hmC, 5-fC and 5-caC | [1, 5] |
| *TET2* | | Tet methylcytosine dioxygenase 2 (ten-eleven translocation-2) | DNA demethylation via conversion of 5-mC to 5-hmC, 5-fC and 5-caC | [1, 5] |
| *TET3* | | Tet methylcytosine dioxygenase 3 (ten-eleven translocation-3) | DNA demethylation via conversion of 5-mC to 5-hmC, 5-fC and 5-caC | [1, 5] |
| *AID* | *AICDA* | Activation-induced cytidine deaminase | DNA demethylation via deamination of 5-mC or 5-hmC; participates in neuronal DNA demethylation | [5-9] |
| *APOBEC1* | | Apolipoprotein B mRNA editing activity DNA deaminase 1 | DNA demethylation via deamination of 5-mC or 5-hmC; participates in neuronal DNA demethylation | [5-7, 10] |
| *APOBEC2* | | Apolipoprotein B mRNA editing activity DNA deaminase 2 | Likely role in DNA demethylation, including potential effects on 5-hmC and possibly 5-mC, however the exact role remains under investigation | [5-8, 10-12] |
| *APOBEC3A* | | Apolipoprotein B mRNA editing activity DNA deaminase 3A | Possible role in DNA demethylation, via deamination of 5-mC or 5-hmC | [5-7, 11] |
| *APOBEC3B* | | Apolipoprotein B mRNA editing activity DNA deaminase 3A | Cytosine deamination with preference for non-methylated cytosine; member of the AID/APOBEC family, other members of which have been linked to DNA demethylation | [7, 13] |
| *APOBEC3C* | | Apolipoprotein B mRNA editing activity DNA deaminase 3C | DNA demethylation, possibly via deamination of 5-mC or 5-hmC | [5-7, 11] |
| *APOBEC3D* | *APOBEC3DE, APOBEC3E* | | Possible role in DNA demethylation | [8, 11, 14] |
| *APOBEC3F* | | Apolipoprotein B mRNA editing activity DNA deaminase 3F | Possible role in DNA demethylation | [8, 14] |
| *APOBEC3G* | | Apolipoprotein B mRNA editing activity DNA deaminase 3G | Possible role in DNA demethylation | [8, 14] |
| *APOBEC3H* | | Apolipoprotein B mRNA editing activity DNA deaminase 3H | Possible role in DNA demethylation | [8] |

| | | | | |
|---|---|---|---|---|
| *APOBEC4* | | Apolipoprotein B mRNA editing activity DNA deaminase C4 | Role in demethylation is unclear as it does not appear to have cytidine deaminating activity | [15, 16] |
| *TDG* | | Thymine-DNA glycosylase | DNA demethylation: recognizes DNA mismatches after 5-mC or 5-hmC deamination, excision of target bases including 5-fC and 5-caC to initiate the BER pathway | [5-7, 17] |
| *UNG* | *UNG1, UDG* | Uracil-DNA glycosylase | DNA demethylation: initiates the BER pathway for removal of uracil residues after dUTP misincorporation or cytosine deamination | [10, 18-20] |
| *SMUG1* | | Single-strand-selective monofunctional uracil-DNA glycosylase | DNA demethylation, DNA repair: member of the uracil-DNA glycosylase superfamily which is involved in DNA repair and DNA demethylation via the BER pathway by participating in degradation of 5-hmU to unmethylated cytosine | [1, 5-7, 21] |
| *GADD45A* | *GADD45* | Growth arrest and DNA damage 45 protein A | DNA demethylation, DNA repair: DNA repair-mediated DNA demethylating factor that can reactivate genes which had been silenced by methylation; also involved in apoptosis | [22-25] |
| *IDH1* | | Isocitrate dehydrogenase 1 | Produces metabolites that interfere with TET-mediated DNA demethylation: IDH1 mutations lead to accumulation of 2-HG, TET inhibition and DNA hypermethylation | [2, 5, 21, 26-29] |
| *IDH2* | | Isocitrate dehydrogenase 2 | Produces metabolites that interfere with TET-mediated DNA demethylation: IDH2 mutations lead to accumulation of 2-HG, TET inhibition and DNA hypermethylation | [2, 5, 21, 26-29] |
| *MBD1* | | Methyl-CpG-binding domain protein 1 | Binding to methylated DNA, transcriptional modulation; transcriptional repression, DNA repair | [1, 30-32] |
| *MBD2* | | Methyl-CpG-binding domain protein 2 | Transcriptional modulation, possible DNA demethylation, binding to methylated DNA | [1, 30, 33] |
| *MBD3* | | Methyl-CpG-binding domain protein 3 | Binding to 5-hmC, transcriptional repression | [1, 30] |
| *MBD4* | *MED1* | Methyl-CpG-binding domain protein 4 | Role in DNA demethylation, DNA mismatch repair, recognizes DNA mismatches after cytosine deamination, possible role in maintenance DNA methylation | [3, 6, 7, 17, 21, 31, 32] |
| *MECP2* | | Methyl-CpG-binding protein 2 | Binding to methylated DNA, binds to DNMT1 and recruits it to hemimethylated DNA for maintenance DNA methylation, participates in TET1 complexes which lead to DNA demethylation, transcriptional repression | [1, 3, 30, 34] |

| | | | | |
|---|---|---|---|---|
| *PCNA* | | Proliferating cell nuclear antigen | Participates in DNA repair and replication; may affect both DNA methylation via interaction with DNMT1 and DNA demethylation by forming a complex with TET1 | [27, 35, 36] |
| *USP7* | *HAUSP* | Herpes virus–associated ubiquitin specific protease | Promotes DNA methylation via control of DNMT1: regulates DNMT1 abundance, stability and activity | [35, 37] |
| *UHRF1* | | Ubiquitin-like, containing plant homeo domain and RING finger 1 | Binds to DNMT1, maintains DNA methylation by targeting DNMT1 to hemimethylated DNA in DNA replication foci; participates in a complex which controls DNMT1 abundance; also binds to DNMT3a, DNMT3b and G9a | [3, 35, 36, 38, 39] |
| *UHRF2* | | Ubiquitin-like, containing plant homeo domain and RING finger 2 | Binds methylated DNA; binds DNMT1, DNMT3a, DNMT3b and G9a | [3, 6, 38] |
| *ZBTB33* | *KAISO* | | Binds methylated DNA, mediates transcriptional repression | [21, 32, 40] |
| *ZBTB4* | | | Binds methylated DNA | [21, 32] |
| *ZBTB38* | | | Binds methylated DNA | [21, 32] |
| *RBPJ* | *RBP-J* | Recombination signal binding protein for immunoglobulin Kappa J | Binds methylated DNA | [41, 42] |
| *KAT5* | *TIP60* | Tat-interactive protein | Interacts with DNMT1 and UHRF1, participates in a complex which controls DNMT1 abundance | [35] |
| *ZSCAN4* | | Zinc finger and SCAN domain containing 4 | Promotes degradation of DNMT1 and UHRF1 in mouse embryonic stem cells, leading to global DNA demethylation | [43] |
| *EHMT1* | *GLP, GLP1* | Euchromatic histone lysine methyltransferase 1 | Maintains imprinting DNA methylation in embryonic stem cells | [44] |
| *EHMT2* | *G9A* | Euchromatic histone lysine methyltransferase 2 | Maintains imprinting DNA methylation in embryonic stem cells | [6, 38, 44] |
| *SETDB1* | *ESET* | | Maintains imprinting DNA methylation in embryonic stem cells by recruiting DNMTs and antagonizing TETs; interacts with DNMT3A and DNMT3B, co-localizes with DNMT3A to promoters silenced in cancer; S*etdb1* knockout in mice leads to minor loss of DNA methylation | [44-46] |
| *ZFP57* | | | Maintains imprinting DNA methylation in embryonic stem cells by recruiting DNMTs and antagonizing TETs | [44] |

| | | | | |
|---|---|---|---|---|
| *DPPA3* | *PGC7, STELLA* | | Maintains imprinting DNA methylation in embryonic stem cells by recruiting DNMTs and antagonizing TETs | [44] |
| *TRIM28* | *KAP1* | | Maintains imprinting DNA methylation in embryonic stem cells by recruiting DNMTs and antagonizing TETs | [44] |
| *EED* | | | PRC2 component, participates in regulation of DNA methylation in mouse embryonic stem cells | [47, 48] |
| *EZH2* | | Enhancer of zeste homolog 2 | Positively regulates DNA methylation in cancer cells, mediates the cross-talk between chromatin states and *de novo* methylation through interaction with DNMTs, participates in regulation of DNA methylation in mouse embryonic stem cells | [4, 47-51] |
| *SUZ12* | | | PRC2 component, participates in regulation of DNA methylation in mouse embryonic stem cells | [47, 48] |
| *KDM2B* | *FBXL10* | | Recruits PRC1 to non-methylated CpGs | [52] |
| *SUV39H1* | | | Binds DNMT1 and DNMT3A, recruits DNMT3A, is associated with DNMT activity | [3, 46, 53] |
| *SUV39H2* | | | Recruits DNMT3A | [46] |
| *CBX1* | *HP1β* | | Binds DNMT1, DNMT3A, is associated with DNMT activity | [53] |
| *DMAP1* | | DNA methyltransferase associated protein 1 | Interacts with DNMT1, activates its methylation activity with a preference for sites of homologous recombination repair | [39, 54] |
| *RNF2* | *RING1B* | | Interacts with MBD1 | [49] |
| *PHC2* | *PH2* | | Interacts with MBD1 | [49] |
| *BMI1* | | | Interacts with DMAP1, found in complexes with DNMT3A/DNMT3B | [49] |
| *HELLS* | *LSH* | | Associates with BMI1 and M33 | [49] |
| *RB1* | *RB* | Retinoblastoma 1 | Recruits DNMT1, stabilizes chromatin | [55] |
| *RBL2* | *RB2, p130* | Retinoblastoma-like 2 | Binds DNMT1 | [36] |
| *HDAC1* | | Histone deacetylase 1 | Interacts with DNMT1; participates in a complex which controls DNMT1 abundance | [35, 56] |
| *CDKL5* | | CDKL5 kinase | Binds DNMT1 and weakly phosphorylates it | [4, 36, 57] |
| *CSNK1D* | *CK1D* | Casein kinase 1δ | Binds DNMT1 and phosphorylates it, reducing its DNA-binding activity | [4, 36, 57] |
| *CSNK1E* | *CK1E* | Casein kinase 1ε | Binds DNMT1 and phosphorylates it, reducing its DNA-binding activity | [36, 57] |
| *CBX2* | *M33* | Chromobox 2 | Found in complexes with DNMT3A/DNMT3B | [49] |
| *CBX4* | | Chromobox 4 | Promotes SUMOulation of DNMT3A | [58] |

| | | | | |
|---|---|---|---|---|
| *SUMO1* | | | SUMOulates DNMT1, DNMT3A, and DNMT3B, increasing DNA methylating activity of DNMT1 and reducing the ability of DNMT3A to interact with other proteins | [4, 59, 60] |
| *SIRT1* | | | Deacetylates DNMT1, modifying its activity | [61] |
| *KMT2A* | *MLL, MLL1, HRX, ALL1* | | *MLL* translocations result in genome-wide hypomethylation in acute myeloid leukemia | [29] |
| *MGMT* | | O(6)-methylguanine-DNA methyltransferase | DNA repair via demethylation of $O^6$-meG; also removes larger $O^6$-alkyl adducts | [62] |
| *ALKBH2* | | AlkB homolog 2 | Demethylates DNA by removing 1-methyladenine and 3-methylcytosine | [63, 64] |
| *ALKBH3* | | AlkB homolog 3 | Demethylates DNA by removing 1-methyladenine and 3-methylcytosine | [63, 64] |
| *BCAT1* | | BCAA transaminase 1 | Restricts αKG levels in AML stem cells, resulting in mutant IDH-like DNA hypermethylation | [65] |

**GMD,** gene affecting DNA methylation or demethylation. The full list abbreviations is provided in the main text.

***Interactions among epigenetic factors affecting DNA methylation***

Many epigenetic factors have complex and intertwined roles affecting DNA methylation. For example, the UDG family members, MBD4, TDG, SMUG1, and UNG, which participate in BER and contribute to DNA demethylation, may have diverse roles and multiple functions [6, 9, 17, 21, 66]. MBD4 is involved in methylation-dependent genome binding and may play a role in the maintenance DNA methylation [3, 26, 32]. MBD1, MBD2, MeCP2, and Kaiso act as transcriptional repressors and DNA-binding proteins interpreting DNA methylation signals, and in addition, MeCP2 participates in binding DNMT1 and in its recruitment to hemimethylated sites for maintenance DNA methylation [3, 32, 34].

There is an extensive cross-talk among the DNA methylation, demethylation, and histone modification pathways in the germ line, embryonic stem cells, normal somatic, and malignant cells [3, 4, 44-46, 49, 53]. For example, histone acetyltransferase SIRT1 deacetylates acetylated DNMT1, modulating its DNA methylating and transcriptional silencing roles [61]. DNMT1 and DNMT3B can bind to histone deacetylases (HDACs), with a direct interaction between HDAC1 and DNMT1 [3, 56]. Histone methyltransferase SUV39H1 can bind DNMT1 and DNMT3A, and both SUV39H1 and SUV39H2 recruit DNMT3A [3, 46, 53]. HP1β (CBX1), which interacts with SUB39H1, binds DNMT1 and DNMT3A and is associated with DNMT activity [53]. Euchromatic histone methyltransferase SETDB1 interacts with DNMT3A and DNMT3B, and its trimethylation of histone H3 lysine 9 (H3K9) plays a role in promoter silencing in cancer in cooperation with DNMT3A [45]. In a complex with KAP1 and ZFP57, SETDB1 also recruits DNMTs to maintain imprinting in embryonic stem cells [44]. Interconnection between chromatin states, histone methylation, and DNMT activities including *de novo* methylation in cancer has been suggested to be mediated by polycomb repressive complexes (PRCs) containing histone methyltransferase EZH2, which has a direct association with DNMT1, DNMT3A, and DNMT3B [4, 49]. Histone lysine demethylase KDM2B recognizes non-methylated CpGs and recruits PRC1 to CpG islands for epigenetic silencing [52]. DNA methylation is influenced by histone modifications, and histone methylation and acetylation marks directly affect DNMT localization, binding, and activities [3, 4, 46, 67].

Some epigenetic factors play important roles in development and in diverse tissues. For example, histone 3 lysine 9 methyltransferase EHMT1 participates in H3K9 dimethylation and trimethylation, in the maintenance of DNA methylation in imprinted regions in embryonic stem cells, and in the regulation of mitochondrial function in mammalian neurons [44, 68]. The histone methyltransferase EHMT2 participates in mono- and dimethylation of H3K9 and in epigenome-wide DNA methylation reprogramming of non-small cell lung cancer, regulating its tumorigenicity and stemness [69].

## References for Table S1


1. Pleyer L, Greil R. Digging deep into "dirty" drugs - modulation of the methylation machinery. Drug Metab Rev. 2015;47:252-79.
2. Suetake I, Shinozaki F, Miyagawa J, Takeshima H, Tajima S. DNMT3L stimulates the DNA methylation activity of Dnmt3a and Dnmt3b through a direct interaction. J Biol Chem. 2004;279:27816-23.
3. Moore LD, Le T, Fan G. DNA methylation and its basic function. Neuropsychopharmacology. 2013;38:23-38.
4. Denis H, Ndlovu MN, Fuks F. Regulation of mammalian DNA methyltransferases: a route to new mechanisms. EMBO Rep. 2011;12:647-56.
5. Shen L, Song CX, He C, Zhang Y. Mechanism and function of oxidative reversal of DNA and RNA methylation. Annu Rev Biochem. 2014;83:585-614.
6. Sadakierska-Chudy A, Kostrzewa RM, Filip M. A comprehensive view of the epigenetic landscape part I: DNA methylation, passive and active DNA demethylation pathways and histone variants. Neurotox Res. 2015;27:84-97.
7. Franchini DM, Petersen-Mahrt SK. AID and APOBEC deaminases: balancing DNA damage in epigenetics and immunity. Epigenomics. 2014;6:427-43.
8. Siriwardena SU, Chen K, Bhagwat AS. Functions and Malfunctions of Mammalian DNA-Cytosine Deaminases: the known knowns and the known unknowns. Chem Rev. 2016;116:12688-710.
9. Dominguez PM, Shaknovich R. Epigenetic function of activation-induced cytidine deaminase and its link to lymphomagenesis. Front Immunol. 2014;5:642.
10. Nabel CS, Jia H, Ye Y, Shen L, Goldschmidt HL, Stivers JT et al. AID/APOBEC deaminases disfavor modified cytosines implicated in DNA demethylation. Nat Chem Biol. 2012;8:751-8.
11. Guo JU, Su Y, Zhong C, Ming GL, Song H. Hydroxylation of 5-methylcytosine by TET1 promotes active DNA demethylation in the adult brain. Cell. 2011;145:423-34.
12. Krzysiak TC, Jung J, Thompson J, Baker D, Gronenborn AM. APOBEC2 is a monomer in solution: implications for APOBEC3G models. Biochemistry. 2012;51:2008-17.
13. Seplyarskiy VB, Soldatov RA, Popadin KY, Antonarakis SE, Bazykin GA, Nikolaev SI. APOBEC-induced mutations in human cancers are strongly enriched on the lagging DNA strand during replication. Genome Res. 2016;26:174-82.
14. Rebhandl S, Huemer M, Greil R, Geisberger R. AID/APOBEC deaminases and cancer. Oncoscience. 2015;2:320-33.
15. Zou J, Wang C, Ma X, Wang E, Peng G. APOBEC3B, a molecular driver of mutagenesis in human cancers. Cell Biosci. 2017;7:29.
16. Marino D, Perkovic M, Hain A, Jaguva Vasudevan AA, Hofmann H, Hanschmann KM et al. APOBEC4 Enhances the Replication of HIV-1. PLoS One. 2016;11:e0155422.
17. Law JA, Jacobsen SE. Establishing, maintaining and modifying DNA methylation patterns in plants and animals. Nat Rev Genet. 2010;11:204-20.
18. Kitamura K, Wang Z, Chowdhury S, Simadu M, Koura M, Muramatsu M. Uracil DNA glycosylase counteracts APOBEC3G-induced hypermutation of hepatitis B viral genomes: excision repair of covalently closed circular DNA. PLoS Pathog. 2013;9:e1003361.
19. Taylor BJ, Nik-Zainal S, Wu YL, Stebbings LA, Raine K, Campbell PJ et al. DNA deaminases induce break-associated mutation showers with implication of APOBEC3B and 3A in breast cancer kataegis. Elife. 2013;2:e00534.
20. Pulukuri SM, Knost JA, Estes N, Rao JS. Small interfering RNA-directed knockdown of uracil DNA glycosylase induces apoptosis and sensitizes human prostate cancer cells to genotoxic stress. Mol Cancer Res. 2009;7:1285-93.
21. Shen H, Laird PW. Interplay between the cancer genome and epigenome. Cell. 2013;153:38-55.
22. Niehrs C, Schafer A. Active DNA demethylation by Gadd45 and DNA repair. Trends Cell Biol. 2012;22:220-7.

23. Schneider-Stock R, Diab-Assef M, Rohrbeck A, Foltzer-Jourdainne C, Boltze C, Hartig R et al. 5-Aza-cytidine is a potent inhibitor of DNA methyltransferase 3a and induces apoptosis in HCT-116 colon cancer cells via Gadd45- and p53-dependent mechanisms. J Pharmacol Exp Ther. 2005;312:525-36.
24. Nguyen AN, Hollenbach PW, Richard N, Luna-Moran A, Brady H, Heise C et al. Azacitidine and decitabine have different mechanisms of action in non-small cell lung cancer cell lines. Lung Cancer: Targets and Therapy. 2010;1:119-40.
25. Barreto G, Schafer A, Marhold J, Stach D, Swaminathan SK, Handa V et al. Gadd45a promotes epigenetic gene activation by repair-mediated DNA demethylation. Nature. 2007;445:671-5.
26. Baubec T, Ivanek R, Lienert F, Schubeler D. Methylation-dependent and -independent genomic targeting principles of the MBD protein family. Cell. 2013;153:480-92.
27. Cartron PF, Nadaradjane A, Lepape F, Lalier L, Gardie B, Vallette FM. Identification of TET1 Partners That Control Its DNA-Demethylating Function. Genes Cancer. 2013;4:235-41.
28. Prensner JR, Chinnaiyan AM. Metabolism unhinged: IDH mutations in cancer. Nat Med. 2011;17:291-3.
29. Schoofs T, Berdel WE, Muller-Tidow C. Origins of aberrant DNA methylation in acute myeloid leukemia. Leukemia. 2014;28:1-14.
30. Das PM, Singal R. DNA methylation and cancer. J Clin Oncol. 2004;22:4632-42.
31. Xu J, Zhu W, Xu W, Cui X, Chen L, Ji S et al. Silencing of MBD1 reverses pancreatic cancer therapy resistance through inhibition of DNA damage repair. Int J Oncol. 2013;42:2046-52.
32. Prokhortchouk E, Hendrich B. Methyl-CpG binding proteins and cancer: are MeCpGs more important than MBDs? Oncogene. 2002;21:5394-9.
33. Cheishvili D, Chik F, Li CC, Bhattacharya B, Suderman M, Arakelian A et al. Synergistic effects of combined DNA methyltransferase inhibition and MBD2 depletion on breast cancer cells; MBD2 depletion blocks 5-aza-2'-deoxycytidine-triggered invasiveness. Carcinogenesis. 2014;35:2436-46.
34. Luo C, Hajkova P, Ecker JR. Dynamic DNA methylation: In the right place at the right time. Science. 2018;361:1336-40.
35. Bronner C. Control of DNMT1 abundance in epigenetic inheritance by acetylation, ubiquitylation, and the histone code. Sci Signal. 2011;4:pe3.
36. Garvilles RG, Hasegawa T, Kimura H, Sharif J, Muto M, Koseki H et al. Dual Functions of the RFTS Domain of Dnmt1 in Replication-Coupled DNA Methylation and in Protection of the Genome from Aberrant Methylation. PLoS One. 2015;10:e0137509.
37. Varol N, Konac E, Bilen CY. Does Wnt/beta-catenin pathway contribute to the stability of DNMT1 expression in urological cancer cell lines? Exp Biol Med (Maywood). 2014.
38. Zhang J, Gao Q, Li P, Liu X, Jia Y, Wu W et al. S phase-dependent interaction with DNMT1 dictates the role of UHRF1 but not UHRF2 in DNA methylation maintenance. Cell Res. 2011;21:1723-39.
39. Cheray M, Pacaud R, Nadaradjane A, Vallette FM, Cartron PF. Specific inhibition of one DNMT1-including complex influences tumor initiation and progression. Clin Epigenetics. 2013;5:9.
40. Walsh CP, Xu GL. Cytosine methylation and DNA repair. Curr Top Microbiol Immunol. 2006;301:283-315.
41. Sadakierska-Chudy A, Filip M. A comprehensive view of the epigenetic landscape. Part II: Histone post-translational modification, nucleosome level, and chromatin regulation by ncRNAs. Neurotox Res. 2015;27:172-97.
42. Bartels SJ, Spruijt CG, Brinkman AB, Jansen PW, Vermeulen M, Stunnenberg HG. A SILAC-based screen for Methyl-CpG binding proteins identifies RBP-J as a DNA methylation and sequence-specific binding protein. PLoS One. 2011;6:e25884.
43. Dan J, Rousseau P, Hardikar S, Veland N, Wong J, Autexier C et al. Zscan4 Inhibits Maintenance DNA Methylation to Facilitate Telomere Elongation in Mouse Embryonic Stem Cells. Cell Rep. 2017;20:1936-49.
44. Zhang T, Termanis A, Ozkan B, Bao XX, Culley J, de Lima Alves F et al. G9a/GLP Complex Maintains Imprinted DNA Methylation in Embryonic Stem Cells. Cell Rep. 2016;15:77-85.

45. Li H, Rauch T, Chen ZX, Szabo PE, Riggs AD, Pfeifer GP. The histone methyltransferase SETDB1 and the DNA methyltransferase DNMT3A interact directly and localize to promoters silenced in cancer cells. J Biol Chem. 2006;281:19489-500.
46. Du J, Johnson LM, Jacobsen SE, Patel DJ. DNA methylation pathways and their crosstalk with histone methylation. Nat Rev Mol Cell Biol. 2015;16:519-32.
47. Smith ZD, Shi J, Gu H, Donaghey J, Clement K, Cacchiarelli D et al. Epigenetic restriction of extraembryonic lineages mirrors the somatic transition to cancer. Nature. 2017;549:543-7.
48. Hagarman JA, Motley MP, Kristjansdottir K, Soloway PD. Coordinate regulation of DNA methylation and H3K27me3 in mouse embryonic stem cells. PLoS One. 2013;8:e53880.
49. Vidal M. Chromatin modifications by polycomb complexes. Epigenetics in biology and medicine. Boca Raton, FL: CRC Press; 2009. p. 131-54.
50. Poirier JT, Gardner EE, Connis N, Moreira AL, de Stanchina E, Hann CL et al. DNA methylation in small cell lung cancer defines distinct disease subtypes and correlates with high expression of EZH2. Oncogene. 2015;34:5869-78.
51. Hoffmann MJ, Engers R, Florl AR, Otte AP, Muller M, Schulz WA. Expression changes in EZH2, but not in BMI-1, SIRT1, DNMT1 or DNMT3B are associated with DNA methylation changes in prostate cancer. Cancer Biol Ther. 2007;6:1403-12.
52. Farcas AM, Blackledge NP, Sudbery I, Long HK, McGouran JF, Rose NR et al. KDM2B links the Polycomb Repressive Complex 1 (PRC1) to recognition of CpG islands. Elife. 2012;1:e00205.
53. Fuks F, Hurd PJ, Deplus R, Kouzarides T. The DNA methyltransferases associate with HP1 and the SUV39H1 histone methyltransferase. Nucleic Acids Res. 2003;31:2305-12.
54. Lee GE, Kim JH, Taylor M, Muller MT. DNA methyltransferase 1-associated protein (DMAP1) is a co-repressor that stimulates DNA methylation globally and locally at sites of double strand break repair. J Biol Chem. 2010;285:37630-40.
55. Ferreira R, Naguibneva I, Pritchard LL, Ait-Si-Ali S, Harel-Bellan A. The Rb/chromatin connection and epigenetic control: opinion. Oncogene. 2001;20:3128-33.
56. Fuks F, Burgers WA, Brehm A, Hughes-Davies L, Kouzarides T. DNA methyltransferase Dnmt1 associates with histone deacetylase activity. Nat Genet. 2000;24:88-91.
57. Sugiyama Y, Hatano N, Sueyoshi N, Suetake I, Tajima S, Kinoshita E et al. The DNA-binding activity of mouse DNA methyltransferase 1 is regulated by phosphorylation with casein kinase 1delta/epsilon. Biochem J. 2010;427:489-97.
58. Li B, Zhou J, Liu P, Hu J, Jin H, Shimono Y et al. Polycomb protein Cbx4 promotes SUMO modification of de novo DNA methyltransferase Dnmt3a. Biochem J. 2007;405:369-78.
59. Lee B, Muller MT. SUMOylation enhances DNA methyltransferase 1 activity. Biochem J. 2009;421:449-61.
60. Cubenas-Potts C, Matunis MJ. SUMO: a multifaceted modifier of chromatin structure and function. Dev Cell. 2013;24:1-12.
61. Peng L, Yuan Z, Ling H, Fukasawa K, Robertson K, Olashaw N et al. SIRT1 deacetylates the DNA methyltransferase 1 (DNMT1) protein and alters its activities. Mol Cell Biol. 2011;31:4720-34.
62. Fahrer J, Kaina B. O6-methylguanine-DNA methyltransferase in the defense against N-nitroso compounds and colorectal cancer. Carcinogenesis. 2013;34:2435-42.
63. Falnes PO, Bjoras M, Aas PA, Sundheim O, Seeberg E. Substrate specificities of bacterial and human AlkB proteins. Nucleic Acids Res. 2004;32:3456-61.
64. Zheng G, Fu Y, He C. Nucleic acid oxidation in DNA damage repair and epigenetics. Chem Rev. 2014;114:4602-20.
65. Raffel S, Falcone M, Kneisel N, Hansson J, Wang W, Lutz C et al. BCAT1 restricts αKG levels in AML stem cells leading to $IDH^{mut}$-like DNA hypermethylation. Nature. 2017;551:384-8.
66. Sjolund AB, Senejani AG, Sweasy JB. MBD4 and TDG: multifaceted DNA glycosylases with ever expanding biological roles. Mutat Res. 2013;743-744:12-25.

67. Weinberg DN, Papillon-Cavanagh S, Chen H, Yue Y, Chen X, Rajagopalan KN et al. The histone mark H3K36me2 recruits DNMT3A and shapes the intergenic DNA methylation landscape. Nature. 2019.
68. Yuan J, Chang SY, Yin SG, Liu ZY, Cheng X, Liu XJ et al. Two conserved epigenetic regulators prevent healthy ageing. Nature. 2020;579:118-22.
69. Pangeni RP, Yang L, Zhang K, Wang J, Li W, Guo C et al. G9a regulates tumorigenicity and stemness through genome-wide DNA methylation reprogramming in non-small cell lung cancer. Clin Epigenetics. 2020;12:88.
