# Supplementary material for: Association of expression of epigenetic molecular factors with DNA methylation and sensitivity to chemotherapeutic agents in cancer cell lines
Source: Clin Epigenetics. 2021 Mar 6;13:49. doi: 10.1186/s13148-021-01026-4 (PMC7936435; doi:10.1186/s13148-021-01026-4)
Supplement: Supplementary file 2 — Additional file 2: Table S2. Numbers of cell lines with available CCLE and GDSC data, analyzed in each cancer category. [file 13148_2021_1026_MOESM2_ESM.pdf]

Table S2. Numbers of cell lines with available CCLE and GDSC data, analyzed in each cancer category

Cancer category	Abbreviation	Number of cell lines
Non-small cell lung cancer	**NSCLC**	96
Breast cancer	**BREAST**	44
Colon adenocarcinoma and rectum adenocarcinoma	**COAD/READ**	43
Melanoma	**MEL**	37
Small cell lung cancer	**SCLC**	36
Glioma brain tumors	**GLIOMA**	36
Mature B-cell lymphoma	**MATBCL**	33
Ovarian cancer	**OVARIAN**	29
Pancreatic adenocarcinoma	**PAAD**	27
Sarcoma	**SAR**	26
Esophageal cancer	**EC**	24
Stomach adenocarcinoma	**STAD**	21
Acute myeloid leukemia	**LAML**	19
Bladder cancer	**BLADDER**	18
Liver hepatocellular carcinoma	**LIHC**	17
Head and neck squamous cell carcinoma	**HNSC**	16
Renal cell carcinoma	**RCC**	15
Chronic lymphocytic leukemia	**CLLE**	15
Multiple myeloma	**MM**	13
Acute lymphocytic leukemia	**ALL**	12
Neuroblastoma	**NB**	11
Uterine corpus endometrial carcinoma	**UCEC**	10
Thyroid carcinoma	**THCA**	10
Chronic myelogenous leukemia	**LCML**	9
Mesothelioma	**MESO**	6
Prostate adenocarcinoma	**PRAD**	6
Hodgkin lymphoma	**HL**	5
Medulloblastoma	**MB**	3
Cervical squamous cell carcinoma and endocervical adenocarcinoma	**CESC**	2
Primitive neuroectodermal tumors	**PNET**	2
Other miscellaneous categories of cancer including rare cancers or cancers with unspecified information	**MISC**	2
T-cell lymphoma	**TCL**	1
Duodenal adenocarcinoma	**DA**	1
