# Supplementary material for: Association of expression of epigenetic molecular factors with DNA methylation and sensitivity to chemotherapeutic agents in cancer cell lines
Source: Clin Epigenetics. 2021 Mar 6;13:49. doi: 10.1186/s13148-021-01026-4 (PMC7936435; doi:10.1186/s13148-021-01026-4)
Supplement: Supplementary file 16 — Additional file 16: Table S16. Significant correlations of GMD expression with drug response in the CCLE-GDSC pancancer dataset from Table 1 which had available data for association analysis in CellminerCDB. [file 13148_2021_1026_MOESM16_ESM.pdf]

Table S16. Significant correlations of GMD expression with drug response in the CCLE-GDSC pancancer dataset from Table 1 which had available data for association analysis in CellminerCDB

Cancer category	GMD	Agent	ρ	p_{FDR}	$r_{CellminerCDB}$	$p_{CellminerCDB}$
Pancancer	*APOBEC3G*	Z-LLNle-CHO	-0.4148	2.64×10^{-9}	-0.12	0.37
Pancancer	*KDM2B*	Quizartinib	-0.4095	8.98×10^{-22}	0.01	0.95
Pancancer	*KDM2B*	Daporinad	-0.4057	7.77×10^{-21}	-0.20	0.15
**Pancancer**	***KDM2B***	**Vorinostat**	**-0.4057**	$\mathbf{2.13 \times 10^{-19}}$	**-0.51**	$\mathbf{4.3 \times 10^{-5}}$
Pancancer	*DNMT1*	Daporinad	-0.4036	1.21×10^{-20}	-0.21	0.11
Pancancer	*KDM2B*	BX-912	-0.4031	3.37×10^{-21}	0.01	0.97
Pancancer	*KDM2B*	I-BET-762	-0.4020	5.08×10^{-21}	-0.15	0.26

All associations satisfied Spearman $|\rho| > 0.4$ and $p_{FDR} < 0.05$ in the CCLE-GDSC pancancer dataset.

ρ, Spearman correlation coefficient for association between GMD expression obtained from CCLE and log(IC50) data available from the GDSC dataset
p_{FDR}**,** FDR adjusted *p*-value for the Spearman correlation between GMD expression and log(IC50) in the CCLE-GDSC dataset
$r_{CellminerCDB}$, Pearson correlation coefficient for association between GMD expression and log(GI50) in the NCI-60 cell lines obtained from CellminerCDB
$p_{CellminerCDB}$, *p*-value for Pearson correlation coefficient for association between GMD expression and log(GI50) in the NCI-60 cell lines obtained from CellminerCDB
The association between *KDM2B* expression and response to vorinostat was statistically significant in both datasets, and it is shown in bold.
