# Supplementary material for: Association of expression of epigenetic molecular factors with DNA methylation and sensitivity to chemotherapeutic agents in cancer cell lines
Source: Clin Epigenetics. 2021 Mar 6;13:49. doi: 10.1186/s13148-021-01026-4 (PMC7936435; doi:10.1186/s13148-021-01026-4)
Supplement: Supplementary file 19 — Additional file 19: Data S1. Selected examples of association of GMD expression with DNA methylation of epigenome targets in individual cancer categories. [file 13148_2021_1026_MOESM19_ESM.pdf]

Data S1

Examples of trans-correlations of GMD expression with DNA methylation of epigenome targets in the pancancer dataset

GMD expression was associated with methylation of the target probes and gene regions in many important cancer genes. For example, *TET3* was positively associated with probe and gene region methylation of *ABL1* and *ABL2*. *ABL2* methylation was also positively correlated with *CBX2, SETDB1,* and *TDG* expression (Tables S4 and S5). *TET3* expression was also associated with methylation of *MET* and *XRCC5* probes (Table S4).

Methylation of *KIFC3* probes was negatively associated with *ZBTB38* expression and positively correlated with expression of *KDM2B, TET3, CBX2, SETDB1, CBX2,* and *TET3* (Table S4). Methylation of *TIMP* probes was positively associated with expression of *TDG, TET3, CBX2,* and *SETDB1*. *PIK3CD* probe methylation was positively correlated with *CBX2* expression. Methylation of a probe in *TGFBI* was negatively correlated with *ZBTB38* expression and positively with *SETDB1* and *KDM2B*. *TGFBI* has been associated with poor prognosis in colorectal cancer and is a predictive biomarker for dasatinib sensitivity [1, 2]. Among the *ABC* family transporter genes, *ABCC1* methylation was positively correlated with *KDM2B* expression, whereas *ABCC3* was negatively correlated with *ZBTB38* and positively with *CBX2, KDM2B, SETDB1, TDG*, and *TET3* (Table S4).

The probe cg13419330 in *IRAK2,* which encodes an activator of the NF-κB pathway, was positively correlated with expression of *CBX2, KDM2B, SETDB1, TDG,* and *TET3,* and negatively with *ZBTB38* expression (Table S4). Methylation of tumor necrosis factor receptor genes was positively correlated with expression of *CBX2* and *KDM2B* for *TNFRSF10B,* and with *KDM2B*, *SETDB1,* and *TET3* for *TNFRSF1A* (Tables S4 and S5). Among the Hippo pathway components, methylation of individual probes and the 5'UTR of *WWTR1* (*TAZ*) were positively correlated with *SETDB1* and *TET3* expression, and a *TEAD1* probe was positively associated with *CBX2* expression (Tables S4 and S5).

Association of GMD expression with methylation of genes encoding other epigenetic factors or involved in transcriptional regulation. GMD expression was significantly correlated with methylation of other genes involved in epigenetic processes or global transcriptional regulation. Expression of the genes encoding histone methyltransferase *SETDB1* and histone lysine demethylase *KDM2B* was positively correlated with methylation of probes in the histone deacetylase *HDAC9* (Table S4), suggesting *HDAC9* regulation by SETB1 and KDM2B or co-regulation among different histone modifiers. *KDM2B* and *TET3* expression was positively associated with probe methylation of *FTO,* which participates in RNA methylation [3]. *TET3* and *SETDB1* expression was positively correlated with methylation of the TSS1500 and a probe in *NNMT* (Tables S4 and S5), whose product regulates the availability of methyl groups for cellular methylation reactions [4, 5].

Methylation of *MED1* and *MED20*, which encode subunits of the mediator of RNA polymerase transcription, was correlated with expression of multiple GMDs (Tables S4 and S5), suggesting links between GMD expression and global transcriptional regulation. Multiple *MED1* probes were positively correlated with *CBX2, SETDB1, TDG,* and *TET3,* and the 5'UTR of *MED1* was also positively correlated with expression of *CBX2* and *TET1*. Methylation of the *MED20* probe cg05689413 was negatively correlated with expression of *ZBTB38* and positively with *CBX2, KDM2B, SETDB1, TDG,* and *TET3.*

Selected examples of trans-correlations of GMD expression with DNA methylation of epigenome targets in individual cancer categories

Examples of epigenetic regulation of cancer-related genes. Methylation of the gene body of *ABL2* was positively associated with *DNMT3A* and *EHMT1* expression in breast cancer (ρ = 0.6445 and 0.6296, p_{FDR} = 0.03643 and 0.0473, respectively; Table S9). The 1st exon of *ABL2* was positively correlated with *HDAC1* expression in COAD/READ (ρ = 0.6873). The 5'UTR and the 1st exon of the tumor suppressor *RUNX1* were positively correlated with *DNMT3A* expression in liver hepatocellular carcinoma (LIHC; ρ = 0.8799 and 0.8750, p_{FDR} = 0.0411 and 0.0460, respectively). Methylation of the upstream region of *MYCN* was negatively correlated with *CBX2* and *TDG* expression in breast cancer and positively correlated with *TDG* and *RNF2* expression in COAD/READ (|ρ| ≥ 0.6339). In CLLE, methylation of the gene body of *EGFR* was strongly positively correlated with *EHMT2* and *PHC2* (ρ = 0.9000).

Examples of associations of methylation of the genes involved in necroptosis and inflammatory signaling. Methylation of gene regions of *MLKL* and *RIPK3* was associated with *CBX1, DNMT3B, IDH2,* and *ZBTB38* expression for *MLKL,* and with *CBX1* and *APOBEC1* expression for *RIPK3* in several tumor categories (Table S9). Methylation of *RIPK2* and *RIPK4* gene regions was also associated with GMD expression. Expression of *DNMT1, DNMT3A, DNMT3B, UHRF1, ZBTB38, APOBEC1, APOBEC3F, HELLS, GADD45A, MBD3, BCAT1, CSNK1D, CSNK1E, EED, CBX2, EHMT2,* and *IDH2* was strongly associated with gene region methylation of multiple components of the TNF-α signaling pathway including *TNF* and other *TNF* family members, e.g., *TNSF11* (*RANKL*) and *TNSF13B* (*BAFF*), *TNFAIP3* and *TNFAIP8L2*, and multiple TNF receptor superfamily members. GMD expression was also associated with methylation of gene regions of *IRAK2, IRAK3,* and *IRAK4*.

Epigenetic regulation of the Hippo signaling pathway. Among the Hippo pathway components (Tables S8 and S9), methylation of the body of *YAP1* was positively correlated with *TET1* expression in stomach adenocarcinoma (ρ = 0.8260, p_{FDR} = 0.0441). Probe and gene region methylation of *WWTR1* (*TAZ*) was positively associated with expression of *CBX1* in stomach adenocarcinoma, *CBX2* in NSCLC, *DNMT3A* and *EHMT1* in breast cancer, *CBX2* and *IDH2* in sarcoma, and *CBX2, UHRF1* and *PHC2* in CLLE (0.6567 ≤ ρ ≤ 0.9357, 0.0131 ≤ p_{FDR} ≤ 0.0372 for gene regions), and negatively correlated with expression of *APOBEC1* in COAD/READ (ρ = -0.6331, p_{FDR} = 0.0490). The body of *TEAD1* was positively correlated with *CBX2* expression in CLLE, whereas methylation of upstream regions of *TEAD2* was negatively correlated with *DMAP2* expression in glioma and positively correlated with expression of *RPBJ* and *UHRF1* in SCLC. The 3'UTR of *LATS1* was positively correlated with *PHC2* in CCLE, while the gene body of *LATS2* was positively associated with *UHRF1* expression in COAD/READ and negatively with *HDAC1* expression in MATBCL. The TSS1500 region of *MST1* was strongly positively correlated with *UHRF1* expression in CLLE (ρ = 0.9536, p_{FDR} = 0.0079). Gene region methylation of *RASSF1, RASSF2, RASSF3, RASSF6, RASSF7,* and *RASSF9* was strongly associated with expression of several GMDs in multiple cancer types.

Epigenetic regulation of genome integrity. GMD expression was significantly associated with methylation of gene regions of *RAD51, RAD51C, RAD50, RAD1, RAD9A, RAD9B, RAD18, RAD21L1,* and *RAD23A*. We observed a negative correlation of methylation of

the TSS1500 of *RAD51* with *DNMT3A* expression in glioma (ρ = -0.6921, p_{FDR} = 0.0399), and many positive associations in CCLE of the TSS200 of *RAD51C* with expression of *EHMT* (ρ = 0.9107, p_{FDR} = 0.0364), and of the TSS200 and the 5'UTR of *RAD50* with expression of *CBX2, SUV39H2,* and *URHF1* ($\rho \geq 0.9321$, $p_{FDR} \leq 0.0201$; Table S9).

Methylation of upstream regions of *XRCC2, XRCC5,* and *XRCC6* was also positively associated with expression of multiple GMDs. For example, in CCLE, the TSS1500 of *XRCC2* was associated with *EHMT2* and *PHC2* (ρ = 0.9286 and 0.9000, respectively), whereas the TSS1500 of *XRCC2* was associated with *EZH2* and *URHF1* (ρ = 0.9071 and 0.9250, respectively). Methylation of gene regions of the tumor suppressor *TP53BP1* was negatively associated with *APOBEC1* and *DMAP1* expression in COAD/READ and bladder cancer and positively correlated with *DNMT1* in SCLC ($|\rho| \geq 0.6829$; Table S9).

Epigenetic regulation of the cGAS-STING pathway. In several cancer categories, increased methylation of gene regions of *TMEM173* (*STING*), *TREX1,* and C6orf150 (*cGAS*), predominantly upstream of these genes, was positively correlated with increased expression of several GMDs (Table S9). TREX1, STING, and cGAS regulate the cytosolic DNA-sensing cGAS-STING innate immune pathway, the activation of which is associated with improved tumor response to drug treatment and immunotherapy [6-12]. In sarcoma, methylation of the TSS200 of *TREX1* was positively correlated with *CBX2* expression (ρ = 0.7915, p_{FDR} = 0.0307). In breast cancer, we observed the positive associations of the TSS200 of *TMEM173* (ρ = 0.6431, p_{FDR} = 0.0374) and of the TSS1500 and the body of *C6orf150* with *EHMT1* expression (ρ = 0.6361, p_{FDR} = 0.0432). In SCLC, methylation of the TSS200, 5'UTR, and the 1st exon of *C6orf150* were associated with *CBX2* (ρ = 0.6968, 0.7338, and 0.7423, p_{FDR} = 0.0364, 0.0197, and 0.0130, respectively). In contrast, methylation of the TSS200 of *TMEM173* was negatively correlated with *APOBEC3C* expression in SCLC (ρ = -0.7192, p_{FDR} = 0.0251). Similarly to individual cancer categories, we also observed significant, albeit weaker ($0.3001 < |\rho| \leq 0.4124$, $7.00 \times 10^{-28} \leq p \leq 6.90 \times 10^{-15}$), associations in the pancancer dataset between methylation of a probe in the TSS200 of *TREX1* with *EHMT2* expression (ρ = 0.4124, 1 $p = 7.00 \times 10^{-28}$) and of upstream gene regions of all three genes with expression of multiple GMDs. These regions included TSS200, 5'UTR, and the 1st exon of *TREX1,* TSS200 of *TMEM173*, and TSS200, 5'UTR, the 1st exon, and the gene body of *C6orf150.* Negative correlations with $\rho < -0.3$ were observed between *TREX1* methylation and expression of *ZBTB38, APOBEC3C,* and *APOBEC3G,* and between *TMEM173* methylation and expression of *APOBEC3C* and *APOBEC3G.* Positive correlations were observed between *TREX1* methylation and expression of *EHMT2, CBX2, KDM2B, SETDB1, EZH2, DNMT1, DNMT3B, TDG, TET1, TET3, BMI1, HELLS, SIRT1, SUV39H2, SUZ12,* and *APOBEC2,* and between methylation of *C6orf150* and expression of *CBX1* and *TET1* (data not shown).

References for Data S1

1. Huang F, Reeves K, Han X, Fairchild C, Platero S, Wong TW et al. Identification of candidate molecular markers predicting sensitivity in solid tumors to dasatinib: rationale for patient selection. Cancer Res. 2007;67:2226-38.
2. Zhu J, Chen X, Liao Z, He C, Hu X. TGFBI protein high expression predicts poor prognosis in colorectal cancer patients. Int J Clin Exp Pathol. 2015;8:702-10.
3. Shen L, Song CX, He C, Zhang Y. Mechanism and function of oxidative reversal of DNA and RNA methylation. Annu Rev Biochem. 2014;83:585-614.
4. Ulanovskaya OA, Zuhl AM, Cravatt BF. NNMT promotes epigenetic remodeling in cancer by creating a metabolic methylation sink. Nat Chem Biol. 2013;9:300-6.
5. Shlomi T, Rabinowitz JD. Metabolism: Cancer mistunes methylation. Nat Chem Biol. 2013;9:293-4.
6. Li T, Chen ZJ. The cGAS-cGAMP-STING pathway connects DNA damage to inflammation, senescence, and cancer. J Exp Med. 2018;215:1287-99.
7. Yan N. Immune Diseases Associated with TREX1 and STING Dysfunction. J Interferon Cytokine Res. 2017;37:198-206.
8. Pantelidou C, Sonzogni O, De Oliveria Taveira M, Mehta AK, Kothari A, Wang D et al. PARP Inhibitor Efficacy Depends on CD8(+) T-cell Recruitment via Intratumoral STING Pathway Activation in BRCA-Deficient Models of Triple-Negative Breast Cancer. Cancer Discov. 2019;9:722-37.
9. Sen T, Rodriguez BL, Chen L, Corte CMD, Morikawa N, Fujimoto J et al. Targeting DNA Damage Response Promotes Antitumor Immunity through STING-Mediated T-cell Activation in Small Cell Lung Cancer. Cancer Discov. 2019;9:646-61.
10. Della Corte CM, Sen T, Gay CM, Ramkumar K, Diao L, Cardnell RJ et al. STING Pathway Expression Identifies NSCLC With an Immune-Responsive Phenotype. J Thorac Oncol. 2020;15:777-91.
11. Naour JL, Zitvogel L, Galluzzi L, Vacchelli E, Kroemer G. Trial watch: STING agonists in cancer therapy. OncoImmunology. 2020; 9:1777624.
12. Wan DS, Jiang W, Hao JW. Research Advances in How the cGAS-STING Pathway Controls the Cellular Inflammatory Response. Frontiers in Immunology. 2020;11.
