# Supplementary figures and images for: Association of expression of epigenetic molecular factors with DNA methylation and sensitivity to chemotherapeutic agents in cancer cell lines
Source: Clin Epigenetics. 2021 Mar 6;13:49. doi: 10.1186/s13148-021-01026-4 (PMC7936435; doi:10.1186/s13148-021-01026-4)

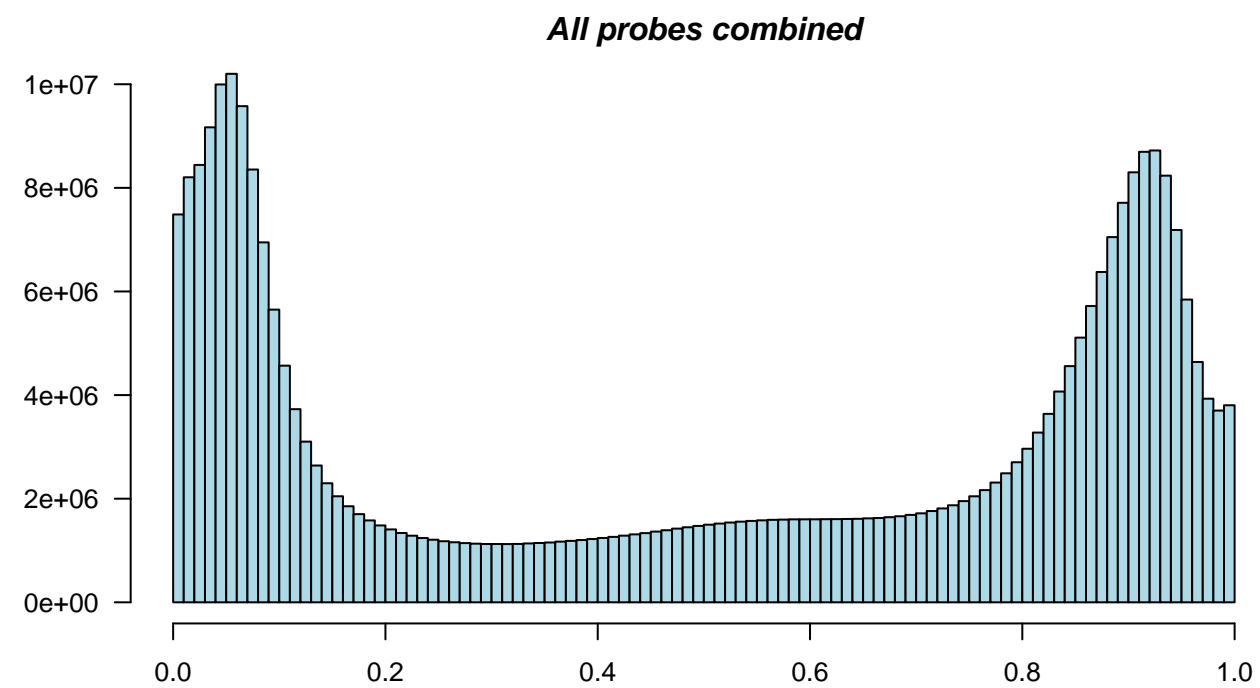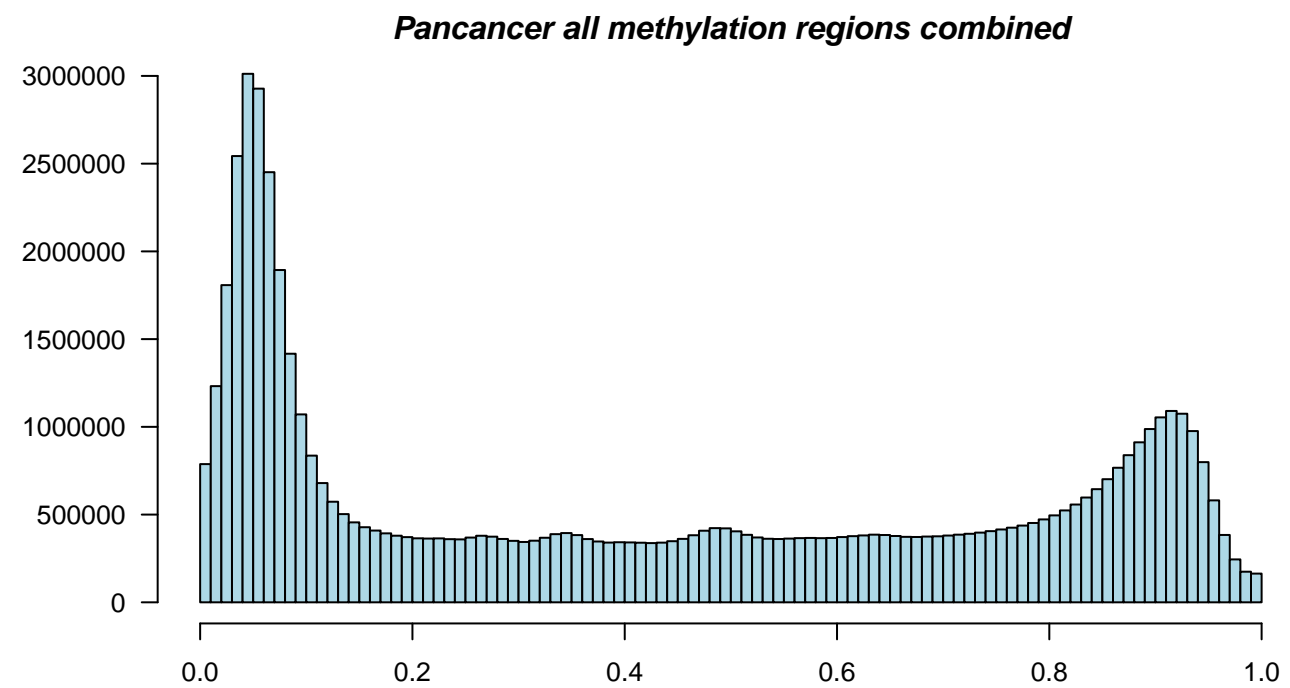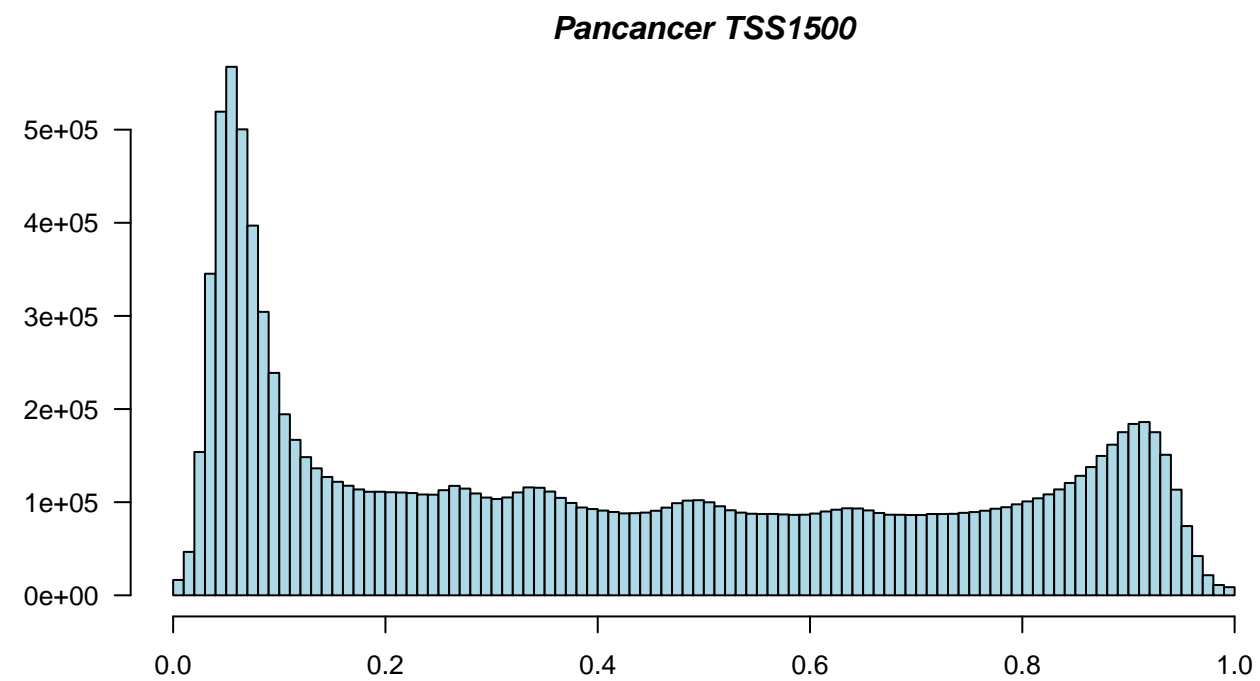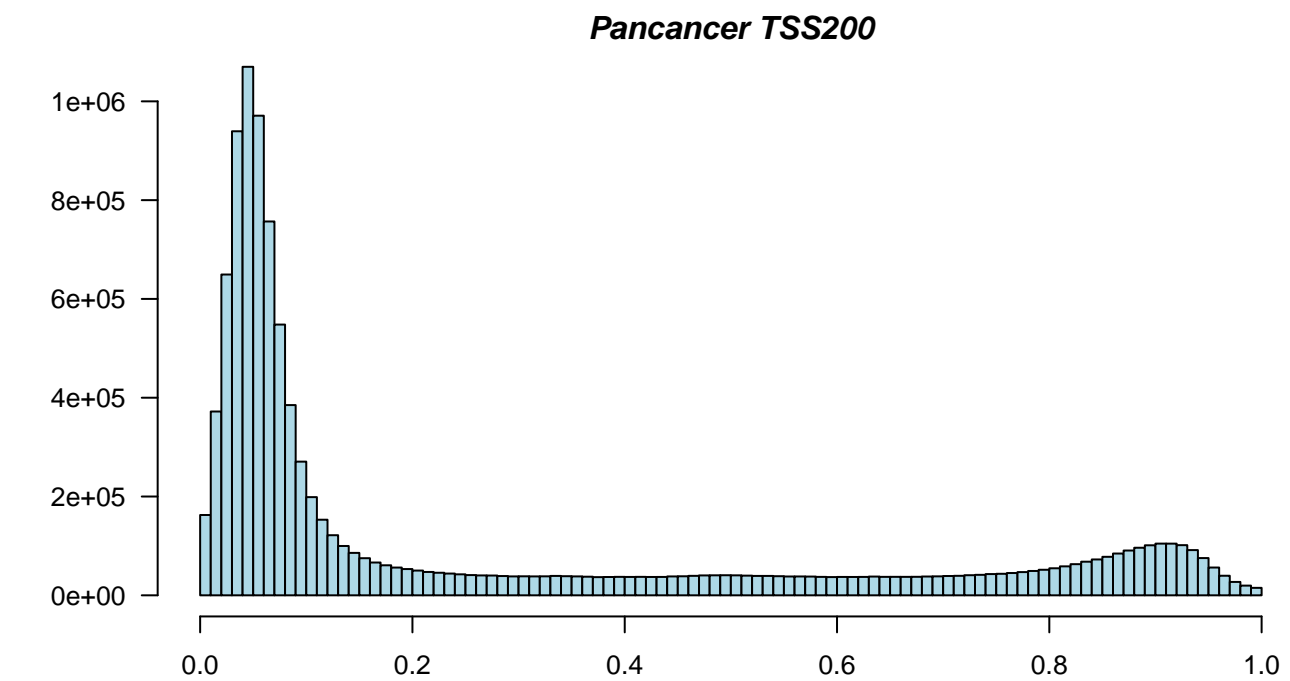

**Fig. S1**

***Pancancer UTR5***

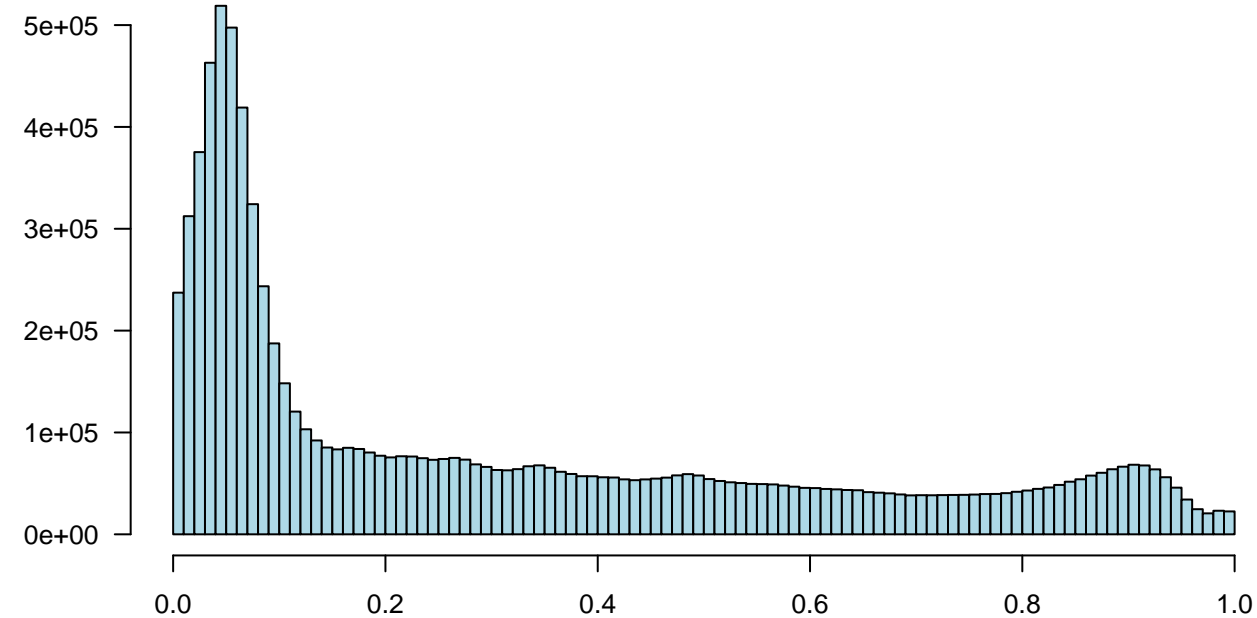

***Pancancer EXON1***

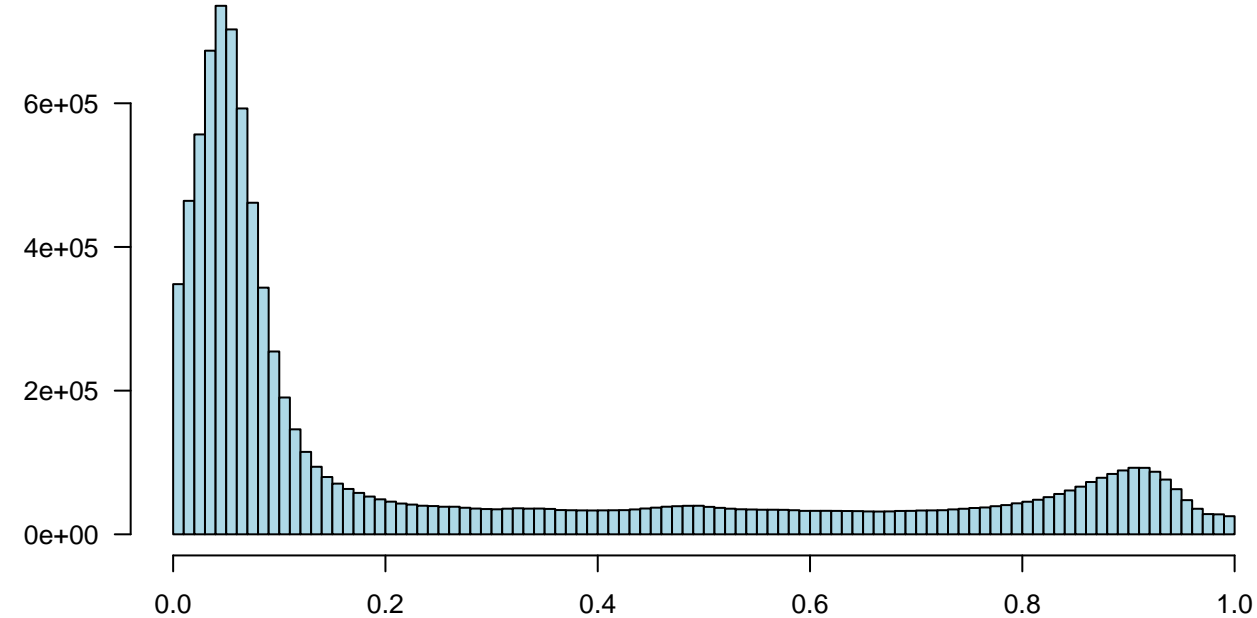

***Pancancer GENE BODY***

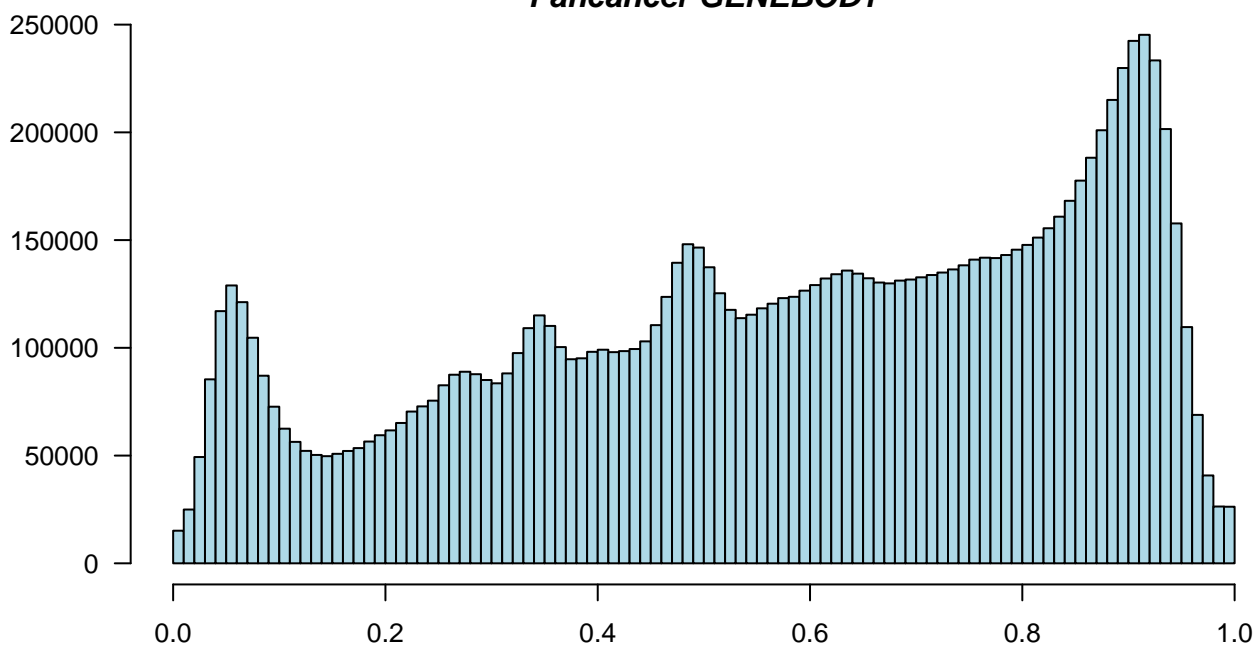

***Pancancer UTR3***

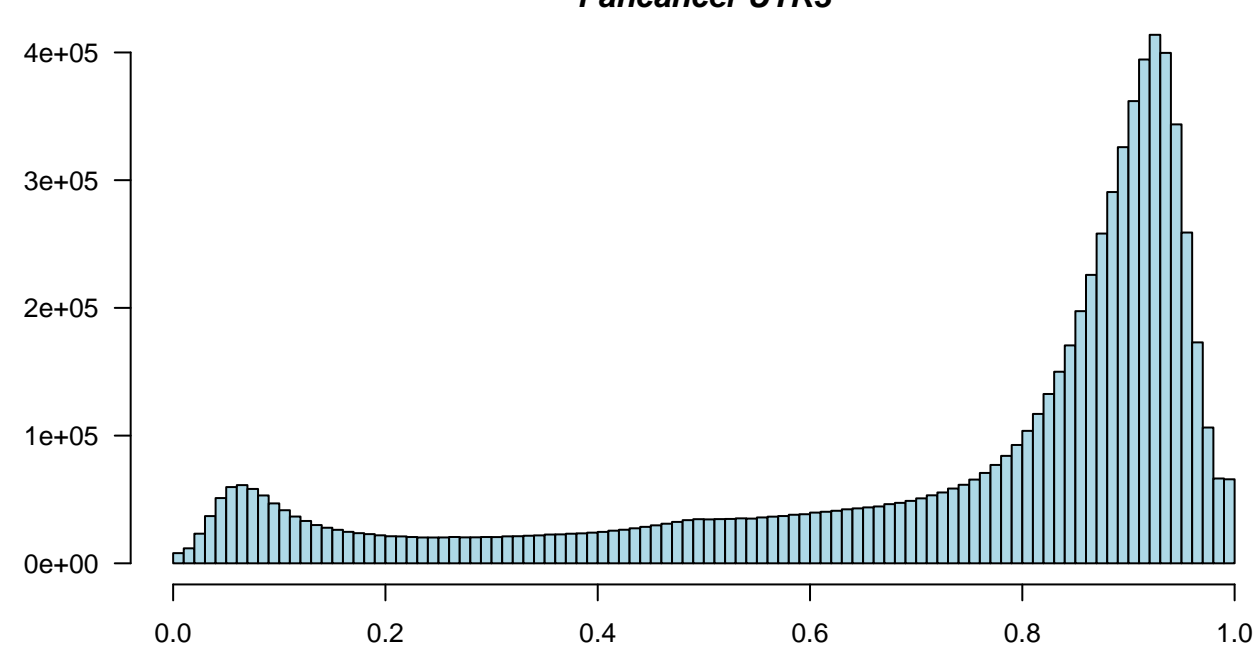

**Fig. S1 (cont.)**

Supplement: Supplementary file 20 — Additional file 20: Figure S1. Plots showing the distribution of DNA methylation values among 424,840 individual probes, the combined distribution of DNA methylation among 93,591 gene regions, and separate distribution for each gene region category in the 645 cell lines in the pancancer dataset. [file 13148_2021_1026_MOESM20_ESM.pdf]

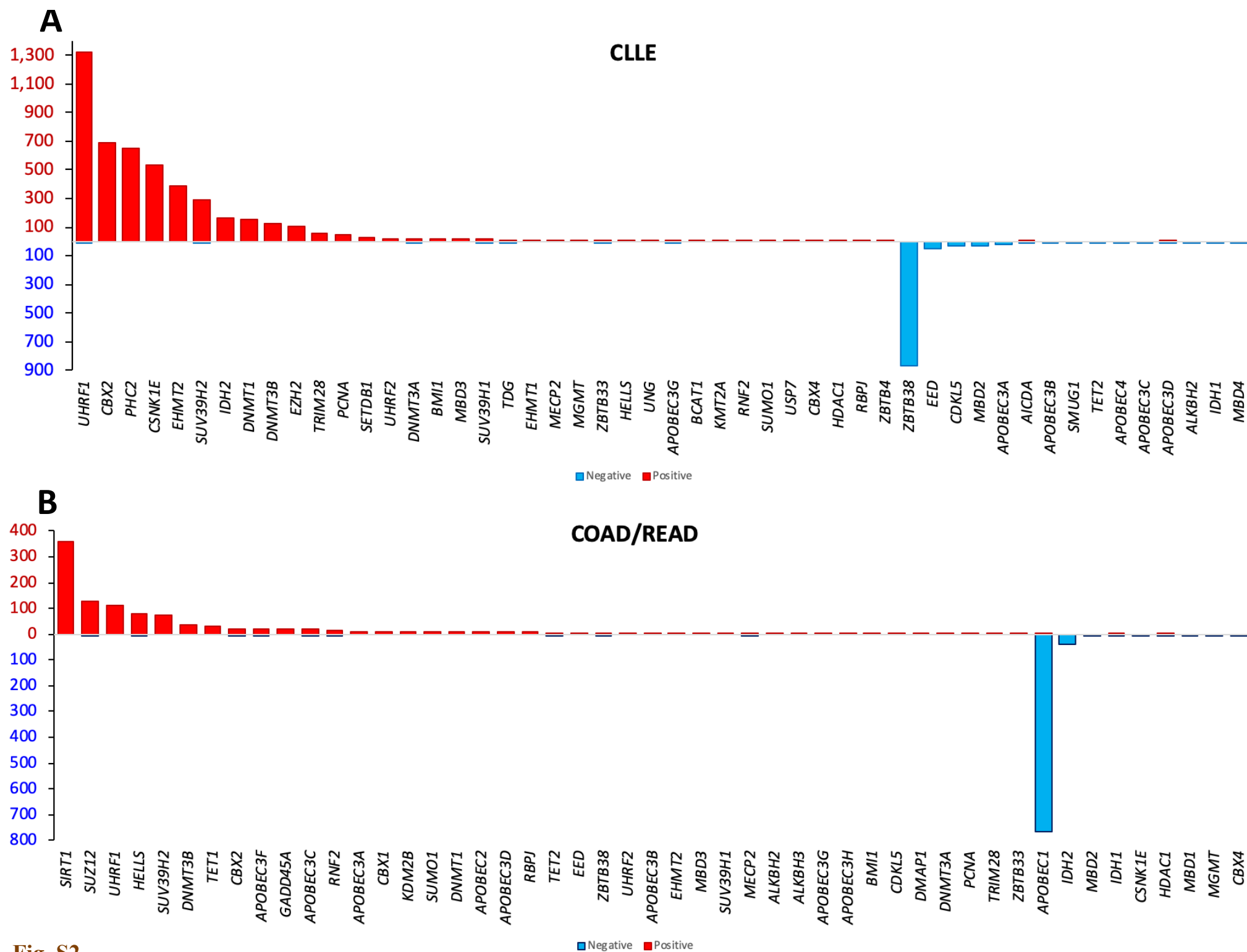

**Fig. S2**

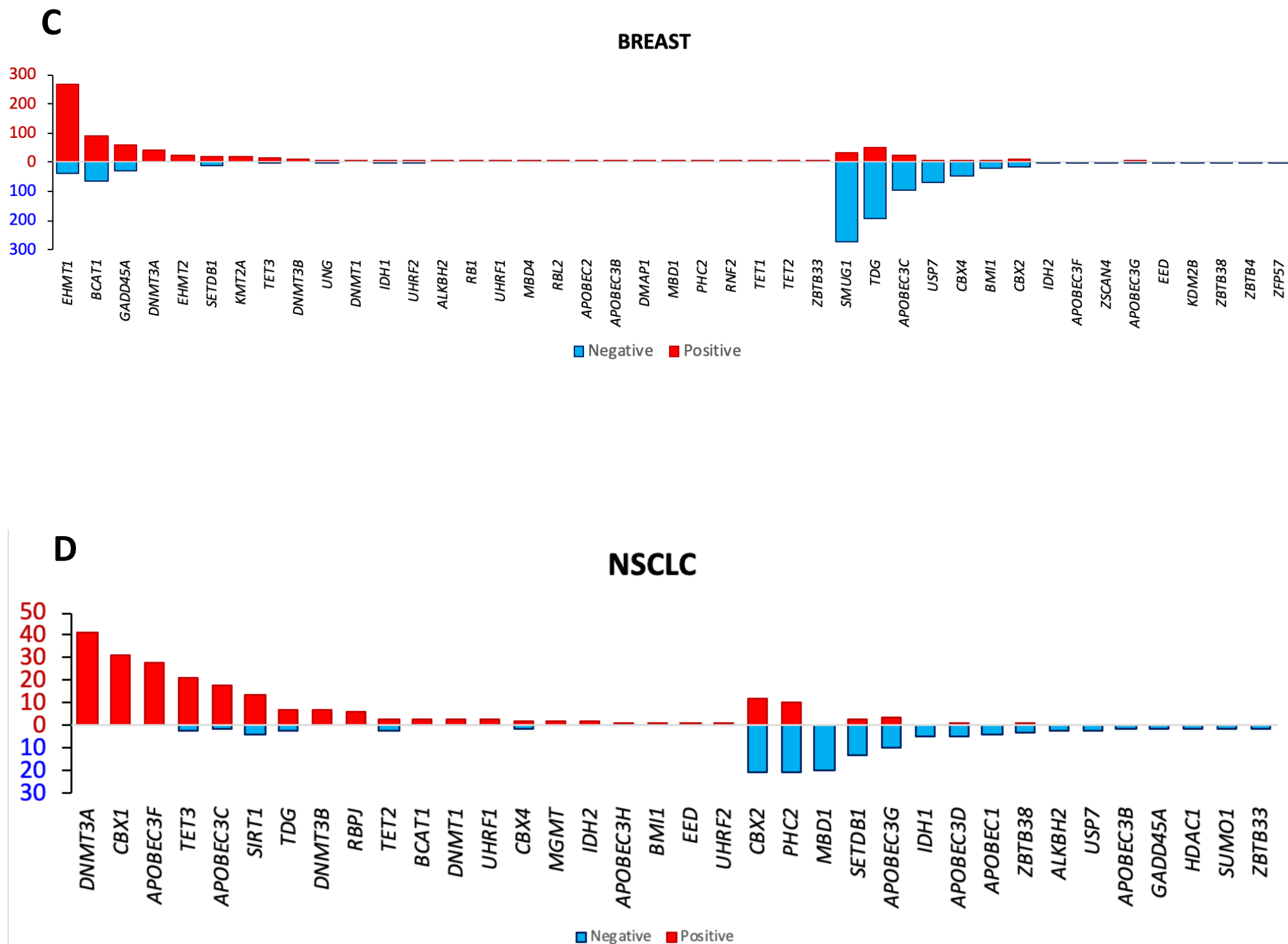

Fig. S2 (cont.)

Supplement: Supplementary file 21 — Additional file 21: Figure S2. A graphical overview of the numbers of strong trans-correlations between GMD expression and methylation of gene regions in selected cancer categories, satisfying pFDR < 0.05 and |ρ| > 0.5. Numbers of trans- and cis-correlations in these and additional cancer categories are provided in Additional file 11: Table S11. Positive correlations are shown as red bars directed upward, whereas negative correlations are shown as blue bars directed downward. (A) Chronic leukocytic leukemia (CLLE). (B) Colon adenocarcinoma and rectum adenocarcinoma (COAD/READ). (C) Breast cancer (BREAST). [file 13148_2021_1026_MOESM21_ESM.pdf]
